# Supplementary material for: The enhancement of activity rescues the establishment of Mecp2 null neuronal phenotypes
Source: EMBO Mol Med. 2021 Mar 5;13(4):e12433. doi: 10.15252/emmm.202012433 (PMC8033520; doi:10.15252/emmm.202012433)

Table of content of the *Appendix*

- **Appendix Table S1**
- **Appendix Table S2**
- **Appendix Table S3**
- **Appendix Figure S1**
- **Appendix Figure S2**
- **Appendix Figure S3**

Appendix Table S1: TaqMan probes and primers for qRT-PCR classified based on biological functions.

A

Transcription factors | Neuron specific | Immediate Early Genes | Ion channels | Gluta and Gaba receptors | Intracellular signaling | Neuroprogenitors

Gene symbol	Gene name	Assay ID
Adcy9	adenylate cyclase 9	Mm00507743_m1
Arc	activity regulated cytoskeletal-associated protein	Mm01204954_g1
Arhgdig	Rho GDP dissociation inhibitor (GDI) gamma	Mm00433576_m1
Bdnf	brain derived neurotrophic factor	Mm04230607_s1
Bhlhb5	basic helix-loop-helix family, member e22	Mm00480708_s1
Btg2	B cell translocation gene 2, anti-proliferative	Mm00476162_m1
Cacna1d	calcium channel, voltage-dependent, L type, alpha 1D	Mm01209927_g1
Camk2b	calcium/calmodulin-dependent protein kinase II, beta	Mm00432284_m1
Camk2d	calcium/calmodulin-dependent protein kinase II, delta	Mm00499266_m1
Ccnd1	cyclin D1	Mm00432359_m1
Clcn3	chloride channel 3	Mm01348786_m1
Clcn7	chloride channel 7	Mm00442400_m1
Cux1	cut-like homeobox 1	Mm01195598_m1
Cux2	cut-like homeobox 2	Mm00500377_m1
Dcx	doublecortin	Mm00438400_m1
Dusp1	dual specificity phosphatase 1	Mm00457274_g1
E2f1	E2F transcription factor 1	Mm00432939_m1
Egr1	early growth response 1	Mm00656724_m1
Fezf2	fez family zinc finger 2	Mm01320619_m1
Gabbr1	gamma-aminobutyric acid (GABA) B receptor, 1	Mm00444578_m1
Gabra3	gamma-aminobutyric acid (GABA) A receptor, subunit alpha	Mm00433489_m1
Gad67	glutamate decarboxylase 1	Mm04207432_g1
Gap43	growth associated protein 43	Mm00500404_m1
Gnaq	guanine nucleotide binding protein, alpha q polypeptide	Mm00492381_m1
Gria1	glutamate receptor, ionotropic, AMPA1 (alpha 1)	Mm00433753_m1
Gria2	glutamate receptor, ionotropic, AMPA2 (alpha 2)	Mm00442822_m1
Gria3	glutamate receptor, ionotropic, AMPA3 (alpha 3)	Mm00497506_m1
Gria4	glutamate receptor, ionotropic, AMPA4 (alpha 4	Mm00444754_m1
Grin1	glutamate receptor, ionotropic, NMDA1 (zeta 1)	Mm00433790_m1
Grin2b	glutamate receptor, ionotropic, NMDA2B (epsilon 2)	Mm00433820_m1
Grin2c	glutamate receptor, ionotropic, NMDA2C (epsilon 3)	Mm00439180_m1
Grin2d	glutamate receptor, ionotropic, NMDA2D (epsilon 4)	Mm00433822_m1
Grm3	glutamate receptor, metabotropic 3	Mm00725298_m1

Gene symbol	Gene name	Assay ID
Grm5	glutamate receptor, metabotropic 5	Mm00690332_m1
Grm7	glutamate receptor, metabotropic 7	Mm01189424_m1
Hes1	hairy and enhancer of split 1 (Drosophila)	Mm01342805_m1
Hes5	hairy and enhancer of split 5 (Drosophila)	Mm00439311_g1
Jph1	junctophilin 1	Mm00517485_m1
Kcc2	solute carrier family 12, member 5	Mm00803929_m1
Kcnq2	potassium voltage-gated channel, subfamily Q, member 2	Mm00440080_m1
Kcnq3	potassium voltage-gated channel, subfamily Q, member 3	Mm00548884_m1
Ki67	antigen identified by monoclonal antibody Ki 67	Mm01278617_m1
Map2	microtubule-associated protein 2	Mm00485231_m1
Nd1	neurogenic differentiation 1	Mm01946604_s1
Necab1	N-terminal EF-hand calcium binding protein 1	Mm00724274_m1
Nestin	nestin	Mm00450205_m1
Neun	RNA binding protein, fox-1 homolog 3	Hs01370654_m1
Ngn2	neurogenin 2	Mm00437603_g1
Nkcc1	solute carrier family 12, member 2	Mm01265951_m1
Notch1	notch 1	Mm00627185_m1
Notch2	notch 2	Mm00803077_m1
Notch3	notch 3	Mm01345646_m1
Nr4a1	nuclear receptor subfamily 4, group A, member 1	Mm01300401_m1
Nr4a2/nurr1	nuclear receptor subfamily 4; group A; member 2	Mm00443060_m1
Pax6	paired box 6	Mm00443081_m1
Plcg2	phospholipase C, gamma 2	Mm01242530_m1
Ppp1r3c	protein phosphatase 1, regulatory (inhibitor) subunit 3C	Mm01204084_m1
Psd95	discs, large homolog 4	Mm00492193_m1
Rest	RE1-silencing transcription factor	Mm00803268_m1
Satb2	special AT-rich sequence binding protein 2	Mm00507331_m1
Scn2b	sodium channel, voltage-gated, type II, beta	Mm01179204_g1
scn3b	sodium channel, voltage-gated, type III, beta	Mm00463369_m1
Scn8a	sodium channel, voltage-gated, type VIII, alpha	Mm00488110_m1
Sox2	SRY (sex determining region Y)-box 2	Mm03053810_s1
Syn1	synapsin I	Mm00449772_m1
Tuj1	tubulin, beta 3 class III	Mm00727586_s1

B

Gene	Sequences	
Arc	FP: GAGCCTACAGAGCCAGGAG	RP: TGCCTTGAAAGTGTCTTGGA
Bdnf (exon 9)	FP: AAGTCTGCATTACATTCCTCGA	RP: GTTTTCTGAAAGAGGGACAGTTT
cFos	FP: AGAGCGCCCCATCCTTAC	RP: GCAGCCATCTTATTCCGTTC
Gabrg2	FP: GCAACCGGAAACCAAGCAAGGATA	RP: GGTGGGTGGCATTGTTCATTTGGA
Gria2	FP: GCCGAGGCGAAACGAATGA	RP: CACTCTCGATGCCATATACGTTG
Gria3	FP: ACCATCAGCATAGGTGGACTT	RP: ACGTGGTAGTTCAAATGGAAGG

Gene	Sequences	
Grin1	FP: AGAGCCCGACCCTAAAAAGAA	RP: CCCTCCTCCCTCTCAATAGC
Grin2a	FP: ACGTGACAGAACGCGAACTT	RP: TCAGTGCGGTTCATCAATAACG
Grin2b	FP: GCCATGAACGAGACTGACCC	RP: GCTTCCTGGTCCGTGTCATC
Kcc2	FP: GCGGGATGCCCAGAAGTCTA	RP: GATGCAAGGCTCCAAACAGAACA
Map2	FP. CTGGACATCAGCCTCACTCA	RP: AATAGGTGCCCTGTGACCTG
Neun	FP: ATCGTAGAGGGACGGAAAATTGA	RP: GTTCCCAGGCTTCTTATTGGTC

Appendix Table S2: List of antibodies and dilutions.

	diliution	manufacturer	code
Gfap	1 : 500	Cell Signaling	GA5
Map2	1 : 500	Cell Signaling	D5G1
Mecp2	1 : 500	Cell Signaling	D4F3
Nestin	1 : 250	Millipore	MAB353
Neun	1 : 500	Cell Signaling	D3S3I
Olig2	1 : 500	AbCam	AB42453
Tuj1	1 : 1000	Sigma Aldrich	T8578

Appendix Table S3: Exact P-value and the statistical test used for each comparison are indicated for each figure.

Figure 1	P value	Comparison	Statistical test
1D	0,566	WT vs KO Div8	Two-way ANOVA and Bonferroni post hoc test
	0,546	WT vs KO Div22	Two-way ANOVA and Bonferroni post hoc test
1G	>0,999	WT vs KO Div8	Two-way ANOVA and Bonferroni post hoc test
	>0,999	WT vs KO Div22	Two-way ANOVA and Bonferroni post hoc test
1J	>0,999	WT vs KO Div8	Two-way ANOVA and Bonferroni post hoc test
	>0,140	WT vs KO Div22	Two-way ANOVA and Bonferroni post hoc test
1L	0,95	WT vs KO Tuj1	Multiple t-test and Holm-Sidak correction for multiple comparison
	0,14	WT vs KO Neun	Multiple t-test and Holm-Sidak correction for multiple comparison
1M	0,001	WT vs KO Avg INT NeuN	Student's t-test
1n'	0,001	WT vs KO Avg INT NeuN	Kolmogorov Smirnov test
1O	>0,999	WT vs KO Div0	Two-way ANOVA and Bonferroni post hoc test
	>0,999	WT vs KO Div3	Two-way ANOVA and Bonferroni post hoc test

	<0,0001	WT vs KO Div14	Two-way ANOVA and Bonferroni post hoc test
1P	<0,0001	WT vs KO Div3 Neurod1	Two-way ANOVA and Sidak correction for multiple comparison
	0,6763	WT vs KO Div14 Neurod1	Two-way ANOVA and Sidak correction for multiple comparison
	0,9825	WT vs KO Div3 Arc	Two-way ANOVA and Sidak correction for multiple comparison
	0,0729	WT vs KO Div14 Arc	Two-way ANOVA and Sidak correction for multiple comparison
	0,9590	WT vs KO Div3 Gria1	Two-way ANOVA and Sidak correction for multiple comparison
	<0,0016	WT vs KO Div14 Gria1	Two-way ANOVA and Sidak correction for multiple comparison
	0,8943	WT vs KO Div3 Grin1	Two-way ANOVA and Sidak correction for multiple comparison
	<0,0041	WT vs KO Div14 Grin1	Two-way ANOVA and Sidak correction for multiple comparison
	0,9788	WT vs KO Div3 Grin2b	Two-way ANOVA and Sidak correction for multiple comparison
	<0,0001	WT vs KO Div14 Grin2b	Two-way ANOVA and Sidak correction for multiple comparison
	0,9953	WT vs KO Div3 Kcc2	Two-way ANOVA and Sidak correction for multiple comparison
	<0,0001	WT vs KO Div14 Kcc2	Two-way ANOVA and Sidak correction for multiple comparison
	0,9078	WT vs KO Div3 Gabra3	Two-way ANOVA and Sidak correction for multiple comparison
	0,0434	WT vs KO Div14 Gabra3	Two-way ANOVA and Sidak correction for multiple comparison
	0,7188	WT vs KO Div3 Psd95	Two-way ANOVA and Sidak correction for multiple comparison
	<0,0001	WT vs KO Div14 Psd95	Two-way ANOVA and Sidak correction for multiple comparison
	0,7223	WT vs KO Div3 Scn2b	Two-way ANOVA and Sidak correction for multiple comparison
	0,0226	WT vs KO Div14 Scn2b	Two-way ANOVA and Sidak correction for multiple comparison
	0,6977	WT vs KO Div3 Syn1	Two-way ANOVA and Sidak correction for multiple comparison
	0,0002	WT vs KO Div14 Syn1	Two-way ANOVA and Sidak correction for multiple comparison
**Figure 2**	**P value**	**Comparison**	**Statistical test**
2C	0,0700	WT vs KO Div18	Two-way ANOVA and Bonferroni post hoc test
	0,0096	WT vs KO Div22	Two-way ANOVA and Bonferroni post hoc test

	0,2000	WT Div18 vs WT Div22 (in time)	Two-way ANOVA and Bonferroni post hoc test
	>0,999	KO Div18 vs KO Div 22 (in time)	Two-way ANOVA and Bonferroni post hoc test
2D	0, 2862	WT vs KO Div18	Two-way ANOVA and Bonferroni post hoc test
	0,0002	WT vs KO Div22	Two-way ANOVA and Bonferroni post hoc test
	0,0013	WT Div18 vs WT Div22 (in time)	Two-way ANOVA and Bonferroni post hoc test
	>0,999	KO Div18 vs KO Div 22 (in time)	Two-way ANOVA and Bonferroni post hoc test
2E	0,2000	WT vs KO Div18	Two-way ANOVA and Bonferroni post hoc test
	0,0100	WT vs KO Div22	Two-way ANOVA and Bonferroni post hoc test
	0,0500	WT Div18 vs WT Div22 (in time)	Two-way ANOVA and Bonferroni post hoc test
	>0,999	KO Div18 vs KO Div 22 (in time)	Two-way ANOVA and Bonferroni post hoc test
2F	>0,999	WT vs KO Div18	Two-way ANOVA and Bonferroni post hoc test
	0,9331	WT vs KO Div22	Two-way ANOVA and Bonferroni post hoc test
	0,046	WT Div18 vs WT Div22 (in time)	Two-way ANOVA and Bonferroni post hoc test
	0,0777	KO Div18 vs KO Div 22 (in time)	Two-way ANOVA and Bonferroni post hoc test
2G	0,2120	WT vs KO Div18	Two-way ANOVA and Bonferroni post hoc test
	0,3691	WT vs KO Div22	Two-way ANOVA and Bonferroni post hoc test
	0,7418	WT Div18 vs WT Div22 (in time)	Two-way ANOVA and Bonferroni post hoc test
	0,4593	KO Div18 vs KO Div 22 (in time)	Two-way ANOVA and Bonferroni post hoc test
2J	0,329	WT vs KO WFMR Ratio	Student's t-test
2K	0,05	WT vs KO WFMR [Hz]	Student's t-test

2M	>0,99	WT vs KO Div3	Two-way ANOVA and Bonferroni post hoc test
	>0,99	WT vs KO Div8	Two-way ANOVA and Bonferroni post hoc test
	0,05	WT vs KO Div14	Two-way ANOVA and Bonferroni post hoc test
	0,01	WT vs KO Div18	Two-way ANOVA and Bonferroni post hoc test
	0,03	WT vs KO Div22	Two-way ANOVA and Bonferroni post hoc test
2N	>0,99	WT vs KO Div3	Two-way ANOVA and Bonferroni post hoc test
	>0,99	WT vs KO Div8	Two-way ANOVA and Bonferroni post hoc test
	0,04	WT vs KO Div14	Two-way ANOVA and Bonferroni post hoc test
	0,13	WT vs KO Div18	Two-way ANOVA and Bonferroni post hoc test
	0,04	WT vs KO Div22	Two-way ANOVA and Bonferroni post hoc test
2O	>0,99	WT vs KO Div3	Two-way ANOVA and Bonferroni post hoc test
	0,02	WT vs KO Div8	Two-way ANOVA and Bonferroni post hoc test
	0,03	WT vs KO Div14	Two-way ANOVA and Bonferroni post hoc test
	0,45	WT vs KO Div18	Two-way ANOVA and Bonferroni post hoc test
	0,41	WT vs KO Div22	Two-way ANOVA and Bonferroni post hoc test
2P	0,10	WT vs KO Div8	Two-way ANOVA and Bonferroni post hoc test
	0,55	WT vs KO Div14	Two-way ANOVA and Bonferroni post hoc test
	0,00	WT vs KO Div18	Two-way ANOVA and Bonferroni post hoc test
	0,01	WT vs KO Div22	Two-way ANOVA and Bonferroni post hoc test
2Q	<0,0001	WT vs KO Div22	Two-way ANOVA and Bonferroni post hoc test
**Figure 3**	**P value**	**Comparison**	**Statistical test**
3B	0,0040	KO vs KO +CX546 Div3-6 Max length	One-way ANOVA and Tukey's post hoc test
	0,0300	KO vs KO +CX546 Div3-6 TDL	One-way ANOVA and Tukey's post hoc test

	0,6930	KO vs KO +CX546 Div3-6 Polarization	One-way ANOVA and Tukey's post hoc test
	0,0120	KO vs KO +CX546 Div3-6 nº of intersections	One-way ANOVA and Tukey's post hoc test
	0,0107	KO vs KO +CX546 Div3-6 Soma size	One-way ANOVA and Tukey's post hoc test
	0,9890	KO vs KO +CX546 Div7-10 Max length	One-way ANOVA and Tukey's post hoc test
	0,0300	KO vs KO +CX546 Div7-10 TDL	One-way ANOVA and Tukey's post hoc test
	0,0930	KO vs KO +CX546 Div7-10 Polarization	One-way ANOVA and Tukey's post hoc test
	0,0004	KO vs KO +CX546 Div7-10 nº of intersections	One-way ANOVA and Tukey's post hoc test
	0,0037	KO vs KO +CX546 Div7-10 Soma size	One-way ANOVA and Tukey's post hoc test
	0,0100	KO +CX546 Div3-6 vs KO +CX546 DIV7-10 Max length	One-way ANOVA and Tukey's post hoc test
	0,9900	KO+CX546 Div3-6 vs KO+CX546 DIV7-10 TDL	One-way ANOVA and Tukey's post hoc test
	0,4627	KO+CX546 Div3-6 vs KO+CX546 DIV7-10Polarization	One-way ANOVA and Tukey's post hoc test
	0,4240	KO+CX546 Div3-6 vs KO+CX546 DIV7-10 nº of intersections	One-way ANOVA and Tukey's post hoc test
	0,6065	KO+CX546 Div3-6 vs KO+CX546 DIV7-10 Soma size	One-way ANOVA and Tukey's post hoc test
3G	<0,0001	KO vs KO+Cx546 Div3-6 Arc	One-way ANOVA and Dunnett's post hoc test
	0,0051	KO vs KO+Cx546 Div3-6 Arhgdig	One-way ANOVA and Dunnett's post hoc test
	0,0228	KO vs KO+Cx546 Div3-6 Bdnf	One-way ANOVA and Dunnett's post hoc test
	0,0064	KO vs KO+Cx546 Div3-6 Cacna1d	One-way ANOVA and Dunnett's post hoc test
	0,0211	KO vs KO+Cx546 Div3-6 Camk2b	One-way ANOVA and Dunnett's post hoc test
	0,3305	KO vs KO+Cx546 Div3-6 Clcn3	One-way ANOVA and Dunnett's post hoc test
	0,0411	KO vs KO+Cx546 Div3-6 Dcx	One-way ANOVA and Dunnett's post hoc test
	0,2991	KO vs KO+Cx546 Div3-6 Gabbr1	One-way ANOVA and Dunnett's post hoc test
	0,7485	KO vs KO+Cx546 Div3-6 Gabra3	One-way ANOVA and Dunnett's post hoc test

	0,0051	KO vs KO+Cx546 Div3-6 Gap43	One-way ANOVA and Dunnett's post hoc test
	0,9395	KO vs KO+Cx546 Div3-6 Gria2	One-way ANOVA and Dunnett's post hoc test
	0,0260	KO vs KO+Cx546 Div3-6 Grin1	One-way ANOVA and Dunnett's post hoc test
	0,5532	KO vs KO+Cx546 Div3-6 Grm5	One-way ANOVA and Dunnett's post hoc test
	0,0005	KO vs KO+Cx546 Div3-6 Kcc2	One-way ANOVA and Dunnett's post hoc test
	0,2557	KO vs KO+Cx546 Div3-6 Kcnq2	One-way ANOVA and Dunnett's post hoc test
	0,0010	KO vs KO+Cx546 Div3-6 Kcnq3	One-way ANOVA and Dunnett's post hoc test
	0,0361	KO vs KO+Cx546 Div3-6 Map2	One-way ANOVA and Dunnett's post hoc test
	0,0265	KO vs KO+Cx546 Div3-6 Neun	One-way ANOVA and Dunnett's post hoc test
	0,0077	KO vs KO+Cx546 Div3-6 Nr4a1	One-way ANOVA and Dunnett's post hoc test
	0,0255	KO vs KO+Cx546 Div3-6 Nurr1	One-way ANOVA and Dunnett's post hoc test
	0,5724	KO vs KO+Cx546 Div3-6 Psd95	One-way ANOVA and Dunnett's post hoc test
	0,8894	KO vs KO+Cx546 Div3-6 Satb2	One-way ANOVA and Dunnett's post hoc test
	0,1364	KO vs KO+Cx546 Div3-6 Scn2b	One-way ANOVA and Dunnett's post hoc test
	0,9045	KO vs KO+Cx546 Div3-6 Scn3b	One-way ANOVA and Dunnett's post hoc test
	0,3100	KO vs KO+Cx546 Div3-6 Scn8a	One-way ANOVA and Dunnett's post hoc test
	0,1787	KO vs KO+Cx546 Div3-6 Syn1	One-way ANOVA and Dunnett's post hoc test
	0,0002	KO vs KO+Cx546 Div3-6 Tuj1	One-way ANOVA and Dunnett's post hoc test
3H	0,6740	KO vs KO+Cx546 Div7-10 Arc	One-way ANOVA and Dunnett's post hoc test
	0,6678	KO vs KO+Cx546 Div7-10 Arhgdig	One-way ANOVA and Dunnett's post hoc test
	0,9862	KO vs KO+Cx546 Div7-10 Bdnf	One-way ANOVA and Dunnett's post hoc test
	0,0360	KO vs KO+Cx546 Div7-10 Cacna1d	One-way ANOVA and Dunnett's post hoc test

	0,5926	KO vs KO+Cx546 Div7-10 Camk2b	One-way ANOVA and Dunnett's post hoc test
	0,9808	KO vs KO+Cx546 Div7-10 Clcn3	One-way ANOVA and Dunnett's post hoc test
	0,5118	KO vs KO+Cx546 Div7-10 Dcx	One-way ANOVA and Dunnett's post hoc test
	0,9814	KO vs KO+Cx546 Div7-10 Gabbr1	One-way ANOVA and Dunnett's post hoc test
	0,3684	KO vs KO+Cx546 Div7-10 Gabra3	One-way ANOVA and Dunnett's post hoc test
	0,5243	KO vs KO+Cx546 Div7-10 Gap43	One-way ANOVA and Dunnett's post hoc test
	0,6163	KO vs KO+Cx546 Div7-10 Gria2	One-way ANOVA and Dunnett's post hoc test
	0,2411	KO vs KO+Cx546 Div7-10 Grin1	One-way ANOVA and Dunnett's post hoc test
	0,8692	KO vs KO+Cx546 Div7-10 Grm5	One-way ANOVA and Dunnett's post hoc test
	0,0294	KO vs KO+Cx546 Div7-10 Kcc2	One-way ANOVA and Dunnett's post hoc test
	0,9232	KO vs KO+Cx546 Div7-10 Kcnq2	One-way ANOVA and Dunnett's post hoc test
	0,1323	KO vs KO+Cx546 Div7-10 Kcnq3	One-way ANOVA and Dunnett's post hoc test
	0,8224	KO vs KO+Cx546 Div7-10 Map2	One-way ANOVA and Dunnett's post hoc test
	0,7530	KO vs KO+Cx546 Div7-10 Neun	One-way ANOVA and Dunnett's post hoc test
	0,6869	KO vs KO+Cx546 Div7-10 Nr4a1	One-way ANOVA and Dunnett's post hoc test
	0,8015	KO vs KO+Cx546 Div7-10 Nurr1	One-way ANOVA and Dunnett's post hoc test
	0,9999	KO vs KO+Cx546 Div7-10 Psd95	One-way ANOVA and Dunnett's post hoc test
	0,6884	KO vs KO+Cx546 Div7-10 Satb2	One-way ANOVA and Dunnett's post hoc test
	0,1788	KO vs KO+Cx546 Div7-10 Scn2b	One-way ANOVA and Dunnett's post hoc test
	0,9483	KO vs KO+Cx546 Div7-10 Scn3b	One-way ANOVA and Dunnett's post hoc test
	0,7654	KO vs KO+Cx546 Div7-10 Scn8a	One-way ANOVA and Dunnett's post hoc test
	>0,9999	KO vs KO+Cx546 Div7-10 Syn1	One-way ANOVA and Dunnett's post hoc test

	0,9668	KO vs KO+Cx546 Div7-10 Tuj1	One-way ANOVA and Dunnett's post hoc test
**Figure 4**	**P value**	**Comparison**	**Statistical test**
4B	> 0,9999	WT vs KO 10	Two-way ANOVA and Bonferroni post hoc test
	> 0,9999	WT vs KO 35	Two-way ANOVA and Bonferroni post hoc test
	0,0002	WT vs KO 60	Two-way ANOVA and Bonferroni post hoc test
	0,211	WT vs KO 85	Two-way ANOVA and Bonferroni post hoc test
	0,2813	WT vs KO 110	Two-way ANOVA and Bonferroni post hoc test
	0,251	WT vs KO 135	Two-way ANOVA and Bonferroni post hoc test
	> 0,9999	WT vs KO 160	Two-way ANOVA and Bonferroni post hoc test
	> 0,9999	WT vs KO 185	Two-way ANOVA and Bonferroni post hoc test
	> 0,9999	WT vs KO 210	Two-way ANOVA and Bonferroni post hoc test
	> 0,9999	WT vs KO 235	Two-way ANOVA and Bonferroni post hoc test
	> 0,9999	WT vs KO +CX564 Div3-6 10	Two-way ANOVA and Bonferroni post hoc test
	> 0,9999	WT vs KO+CX546 Div3-6 35	Two-way ANOVA and Bonferroni post hoc test
	> 0,9999	WT vs KO +CX546 Div3-6 60	Two-way ANOVA and Bonferroni post hoc test
	> 0,9999	WT vs KO+CX546 Div3-6 85	Two-way ANOVA and Bonferroni post hoc test
	> 0,9999	WT vs KO+CX546 Div3-6 110	Two-way ANOVA and Bonferroni post hoc test
	0,7362	WT vs KO +CX546 Div3-6 135	Two-way ANOVA and Bonferroni post hoc test
	0,6316	WT vs KO +CX546 Div3-6160	Two-way ANOVA and Bonferroni post hoc test
	> 0,9999	WT vs KO+CX546 Div3-6 185	Two-way ANOVA and Bonferroni post hoc test
	> 0,9999	WT vs KO+CX546 Div3-6210	Two-way ANOVA and Bonferroni post hoc test
	> 0,9999	WT vs KO+CX546 Div3-6 235	Two-way ANOVA and Bonferroni post hoc test
	> 0,9999	KO vs KO+CX546 Div3-6 10	Two-way ANOVA and Bonferroni post hoc test

	> 0,9999	KO vs KO +CX546 Div3-6 35	Two-way ANOVA and Bonferroni post hoc test
	0,0034	KO vs KO+CX546 Div3-6 60	Two-way ANOVA and Bonferroni post hoc test
	> 0,9999	KO vs KO+CX546 Div3-6 85	Two-way ANOVA and Bonferroni post hoc test
	0,2676	KO vs KO+CX546 Div3-6 110	Two-way ANOVA and Bonferroni post hoc test
	> 0,9999	KO vs KO+CX546 Div3-6 135	Two-way ANOVA and Bonferroni post hoc test
	> 0,9999	KO vs KO+CX546 Div3-6160	Two-way ANOVA and Bonferroni post hoc test
	> 0,9999	KO vs KO+CX546 Div3-6185	Two-way ANOVA and Bonferroni post hoc test
	> 0,9999	KO vs KO +CX546 Div3-6 210	Two-way ANOVA and Bonferroni post hoc test
	> 0,9999	KO vs KO+CX546 Div3-6 235	Two-way ANOVA and Bonferroni post hoc test
4C	> 0,9999	WT vs KO 10	Two-way ANOVA and Bonferroni post hoc test
	> 0,9999	WT vs KO 35	Two-way ANOVA and Bonferroni post hoc test
	0,0004	WT vs KO 60	Two-way ANOVA and Bonferroni post hoc test
	0,2539	WT vs KO 85	Two-way ANOVA and Bonferroni post hoc test
	0,331	WT vs KO 110	Two-way ANOVA and Bonferroni post hoc test
	0,2981	WT vs KO 135	Two-way ANOVA and Bonferroni post hoc test
	> 0,9999	WT vs KO 160	Two-way ANOVA and Bonferroni post hoc test
	> 0,9999	WT vs KO 185	Two-way ANOVA and Bonferroni post hoc test
	> 0,9999	WT vs KO 210	Two-way ANOVA and Bonferroni post hoc test
	> 0,9999	WT vs KO 235	Two-way ANOVA and Bonferroni post hoc test
	> 0,9999	WT vs KO+CX546 Div7-10 10	Two-way ANOVA and Bonferroni post hoc test
	> 0,9999	WT vs KO+CX546 Div7-10 35	Two-way ANOVA and Bonferroni post hoc test
	< 0,0001	WT vs KO+CX546 Div7-10 60	Two-way ANOVA and Bonferroni post hoc test

	> 0,9999	WT vs KO+CX546 Div7-10 85	Two-way ANOVA and Bonferroni post hoc test
	0,0261	WT vs KO+CX546 Div7-10 110	Two-way ANOVA and Bonferroni post hoc test
	0,0265	WT vs KO+CX546 Div7-10 135	Two-way ANOVA and Bonferroni post hoc test
	> 0,9999	WT vs KO+CX546 Div7-10 160	Two-way ANOVA and Bonferroni post hoc test
	> 0,9999	WT vs KO+CX546 Div7-10 185	Two-way ANOVA and Bonferroni post hoc test
	> 0,9999	WT vs KO +CX546 Div7-10 210	Two-way ANOVA and Bonferroni post hoc test
	> 0,9999	WT vs KO+CX546 Div7-10 235	Two-way ANOVA and Bonferroni post hoc test
	> 0,9999	KO vs KO+CX546 Div7-1010	Two-way ANOVA and Bonferroni post hoc test
	> 0,9999	KO vs KO+CX546 Div7-10 35	Two-way ANOVA and Bonferroni post hoc test
	0,1093	KO vs KO+CX546 Div7-10 60	Two-way ANOVA and Bonferroni post hoc test
	0,595	KO vs KO+CX546 Div7-10 85	Two-way ANOVA and Bonferroni post hoc test
	0,6439	KO vs KO+CX546 Div7-10 110	Two-way ANOVA and Bonferroni post hoc test
	0,6995	KO vs KO+CX546 Div7-10 135	Two-way ANOVA and Bonferroni post hoc test
	> 0,9999	KO vs KO+CX546 Div7-10 160	Two-way ANOVA and Bonferroni post hoc test
	> 0,9999	KO vs KO+CX546 Div7-10 185	Two-way ANOVA and Bonferroni post hoc test
	> 0,9999	KO vs KO+CX546 Div7-10 210	Two-way ANOVA and Bonferroni post hoc test
	> 0,9999	KO vs KO+CX546 Div7-10 235	Two-way ANOVA and Bonferroni post hoc test
4E	0,0026	WT vs KO Div3	Two-way ANOVA and Bonferroni post hoc test
	<0,0001	WT vs KO Div6	Two-way ANOVA and Bonferroni post hoc test
	<0,0001	WT vs KO Div9	Two-way ANOVA and Bonferroni post hoc test
4F	<0,0001	WT vs KO	One-way ANOVA and Tukey's post hoc test
	0,06	WT vs KO+Cx546 Div3-6	One-way ANOVA and Tukey's post hoc test

	<0,0001	KO vs KO+CX546 Div7-10	One-way ANOVA and Tukey's post hoc test
4G	<0,0001	WT vs KO	One-way ANOVA and Tukey's post hoc test
	<0,0001	WT vs KO+Cx546 Div3-6	One-way ANOVA and Tukey's post hoc test
	>0,999	KO vs KO+CX546 Div7-10	One-way ANOVA and Tukey's post hoc test
4I	0,3447	KO vs KO+CX546 Camk2b	One-way ANOVA and Tukey's post hoc test
	0,023	KO vs KO+CX546 Gabbr1	One-way ANOVA and Tukey's post hoc test
	0,0346	KO vs KO+CX546 Gap43	One-way ANOVA and Tukey's post hoc test
	0,0365	KO vs KO+CX546 Grin1	One-way ANOVA and Tukey's post hoc test
	0,0005	KO vs KO+CX546 Kcc2	One-way ANOVA and Tukey's post hoc test
	0,0152	KO vs KO+CX546 Rest	One-way ANOVA and Tukey's post hoc test
	0,1561	KO vs KO+CX546 Scn2b	One-way ANOVA and Tukey's post hoc test
	0,1053	KO vs KO+CX546 Scn3b	One-way ANOVA and Tukey's post hoc test
	0,0277	KO vs KO+CX546 Tuj1	One-way ANOVA and Tukey's post hoc test
	0,6368	KO vs KO+CX546+Nif Camk2b	One-way ANOVA and Tukey's post hoc test
	0,5597	KO vs KO+CX546+Nif Gabbr1	One-way ANOVA and Tukey's post hoc test
	0,0016	KO vs KO+CX546+Nif Gap43	One-way ANOVA and Tukey's post hoc test
	0,6612	KO vs KO+CX546+Nif Grin1	One-way ANOVA and Tukey's post hoc test
	0,815	KO vs KO+CX546+Nif Kcc2	One-way ANOVA and Tukey's post hoc test
	0,7162	KO vs KO+CX546+Nif Rest	One-way ANOVA and Tukey's post hoc test
	0,6096	KO vs KO+CX546+Nif Scn2b	One-way ANOVA and Tukey's post hoc test
	0,0624	KO vs KO+CX546+Nif Scn3b	One-way ANOVA and Tukey's post hoc test
	0,9287	KO vs KO+CX546+Nif Tuj1	One-way ANOVA and Tukey's post hoc test

	0,0498	KO+CX546 vs KO+CX546+Nif Camk2b	One-way ANOVA and Tukey's post hoc test
	0,0007	KO+CX546 vs KO+CX546+Nif Gabbr1	One-way ANOVA and Tukey's post hoc test
	0,2113	KO+CX546 vs KO+CX546+Nif Gap43	One-way ANOVA and Tukey's post hoc test
	0,0031	KO+CX546 vs KO+CX546+Nif Grin1	One-way ANOVA and Tukey's post hoc test
	0,0031	KO+CX546 vs KO+CX546+Nif Kcc2	One-way ANOVA and Tukey's post hoc test
	0,0902	KO+CX546 vs KO+CX546+Nif Rest	One-way ANOVA and Tukey's post hoc test
	0,0111	KO+CX546 vs KO+CX546+Nif Scn2b	One-way ANOVA and Tukey's post hoc test
	0,8402	KO+CX546 vs KO+CX546+Nif Scn3b	One-way ANOVA and Tukey's post hoc test
	0,01	KO+CX546 vs KO+CX546+NIf Tuj1	One-way ANOVA and Tukey's post hoc test
4L	0,0067	KO vs KO+CX546	One-way ANOVA and Tukey's post hoc test
	0,9945	KO vs KO+CX546+Nif	One-way ANOVA and Tukey's post hoc test
	0,009	KO CX vs KO+CX546+Nif	One-way ANOVA and Tukey's post hoc test
**Figure 5**	**P value**	**Comparison**	**Statistical test**
5B	0,002	KO vs KO+CX546	Gehan-Breslow-Wilcoxon test
5C	< 0,0001	WT vs KO P30	Two-way ANOVA and Bonferroni post hoc test
	0,0002	WT vs KO P50	Two-way ANOVA and Bonferroni post hoc test
	0,004	WT vs KO P70	Two-way ANOVA and Bonferroni post hoc test
	0,9759	WT vs KO P90	Two-way ANOVA and Bonferroni post hoc test
	0,4899	WT vs KO+CX546 P30	Two-way ANOVA and Bonferroni post hoc test
	0,1828	WT vs KO+CX546 P50	Two-way ANOVA and Bonferroni post hoc test
	0,9989	WT vs KO+CX546 P70	Two-way ANOVA and Bonferroni post hoc test
	0,563	WT vs KO+CX546 P90	Two-way ANOVA and Bonferroni post hoc test
	0,0013	KO vs KO+CX546 P30	Two-way ANOVA and Bonferroni post hoc test

	< 0,0001	KO vs KO+CX546 P50	Two-way ANOVA and Bonferroni post hoc test
	0,0018	KO vs KO+CX546 P70	Two-way ANOVA and Bonferroni post hoc test
	0,8901	KO vs KO+CX546 P90	Two-way ANOVA and Bonferroni post hoc test
5F	0,0082	WT vs KO time1	Two-way ANOVA and Bonferroni post hoc test
	0,0195	WT vs KO time2	Two-way ANOVA and Bonferroni post hoc test
	0,0799	WT vs KO time3	Two-way ANOVA and Bonferroni post hoc test
	0,0033	WT vs KO+CX546 time1	Two-way ANOVA and Bonferroni post hoc test
	0,241	WT vs KO+CX546 time2	Two-way ANOVA and Bonferroni post hoc test
	0,7582	WT vs KO+CX546 time3	Two-way ANOVA and Bonferroni post hoc test
	0,5645	KO time 1 vs time 2	Two-way ANOVA and Bonferroni post hoc test
	0,1088	KO time 1 vs time 3	Two-way ANOVA and Bonferroni post hoc test
	>0,9999	KO time 2 vs time 3	Two-way ANOVA and Bonferroni post hoc test
	0,0319	KO+CX546 time 1 vs time 2	Two-way ANOVA and Bonferroni post hoc test
	0,0025	KO+CX546 time 1 vs time 3	Two-way ANOVA and Bonferroni post hoc test
	>0,9999	KO+CX546 time 2 vs time 3	Two-way ANOVA and Bonferroni post hoc test
	0,7693	WT time 1 vs time 2	Two-way ANOVA and Bonferroni post hoc test
	0,3885	WT time 1 vs time 3	Two-way ANOVA and Bonferroni post hoc test
	>0,9999	WT time 2 vs time 3	Two-way ANOVA and Bonferroni post hoc test
5G	0,0184	WT vs KO	One-way ANOVA and Tukey's post hoc test
	0,0732	WT vs KO+CX546	One-way ANOVA and Tukey's post hoc test
	0,676	KO vs KO+CX546	One-way ANOVA and Tukey's post hoc test
5H	0,069	WT vs KO	One-way ANOVA and Tukey's post hoc test

	0,6252	WT vs KO+CX546	One-way ANOVA and Tukey's post hoc test
5I	0,0071	WT vs KO	One-way ANOVA and Tukey's post hoc test
	0,5672	WT vs KO+CX546	One-way ANOVA and Tukey's post hoc test
5J	0,0472	WT vs KO	One-way ANOVA and Tukey's post hoc test
	0,9809	WT vs KO+CX546	One-way ANOVA and Tukey's post hoc test
**Figure EV1**	**P value**	**Comparison**	**Statistical test**
EV1 C	0,084	WT vs KO Div8	Student's t-test
EV1 D	<0,0001	DIV0 vs. DIV3	One-way ANOVA and Tukey's post hoc test
	<0,0001	DIV0 vs. DIV8	One-way ANOVA and Tukey's post hoc test
	<0,0001	DIV0 vs. DIV14	One-way ANOVA and Tukey's post hoc test
	0,5731	DIV3 vs. DIV8	One-way ANOVA and Tukey's post hoc test
	0,0011	DIV3 vs. DIV14	One-way ANOVA and Tukey's post hoc test
	0,0702	DIV8 vs. DIV14	One-way ANOVA and Tukey's post hoc test
EV1 F	0,485	Tuj1 and Mecp2 positive vs Tuj1 and Mecp2 negative	Student's t-test
	0,2155	Gfap and Mecp2 positive vs Gfap and Mecp2 negative	Student's t-test
**Figure EV2**	**P value**	**Comparison**	**Statistical test**
EV2 B	1,456E-05	WT vs KO Gria2	Multiple t-test and Holm-Sidak correction for multiple comparison
	0,0092352	WT vs KO Gria3	Multiple t-test and Holm-Sidak correction for multiple comparison
	0,0628577	WT vs KO Grin1	Multiple t-test and Holm-Sidak correction for multiple comparison
	0,0006896	WT vs KO Grin2a	Multiple t-test and Holm-Sidak correction for multiple comparison
	0,0079372	WT vs KO Grin2b	Multiple t-test and Holm-Sidak correction for multiple comparison
	0,0623571	WT vs KO Gabrg2	Multiple t-test and Holm-Sidak correction for multiple comparison
	0,0145598	WT vs KO Arc	Multiple t-test and Holm-Sidak correction for multiple comparison

	0,513755	WT vs KO cFos	Multiple t-test and Holm-Sidak correction for multiple comparison
	0,0002487	WT vs KO Neun	Multiple t-test and Holm-Sidak correction for multiple comparison
	0,0671653	WT vs KO Map2	Multiple t-test and Holm-Sidak correction for multiple comparison
**Figure EV3**	**P value**	**Comparison**	**Statistical test**
EV3 A	>0,9999	WT vs KO Div4	Two-way ANOVA and Bonferroni post hoc test
	>0,9999	WT vs KO Div8	Two-way ANOVA and Bonferroni post hoc test
	0,005	WT vs KO Div14	Two-way ANOVA and Bonferroni post hoc test
EV3 B	0,05	WT vs WT+CX546	One-way ANOVA and Tukey's post hoc test
	0,8471	WT vs WT+CX546+Nbqx	One-way ANOVA and Tukey's post hoc test
	0,0261	WT+CX546 vs WT+CX546+Nbqx	One-way ANOVA and Tukey's post hoc test
**Figure EV4**	**P value**	**Comparison**	**Statistical test**
EV4 A	0,0021	WT vs KO	One-way ANOVA and Tukey's post hoc test
	0,8886	WT vs KO+KCl 4mM	One-way ANOVA and Tukey's post hoc test
	0,0079	KO vs KO+KCL 4mM	One-way ANOVA and Tukey's post hoc test
EV4 B	0,0249	WT vs KO	One-way ANOVA and Tukey's post hoc test
	0,7201	WT vs KO+KCl 4mM	One-way ANOVA and Tukey's post hoc test
	0,0876	KO vs KO+KCL 4mM	One-way ANOVA and Tukey's post hoc test
EV4 C	0,2023	WT vs KO	One-way ANOVA and Tukey's post hoc test
	0,1207	WT vs KO+KCl 4mM	One-way ANOVA and Tukey's post hoc test
	0,0018	KO vs KO+KCL 4mM	One-way ANOVA and Tukey's post hoc test
EV4 D	0,0044	WT vs KO	One-way ANOVA and Tukey's post hoc test
	0,0708	WT vs KO+KCl 4mM	One-way ANOVA and Tukey's post hoc test
	0,2834	KO vs KO+KCL 4mM	One-way ANOVA and Tukey's post hoc test

EV4 F	0,0001	WT vs KO	Two-way ANOVA and Bonferroni post hoc test
	> 0,9999	WT+KCL vs KO+KCl div 0-7	Two-way ANOVA and Bonferroni post hoc test
	0,05	WT+KCL vs KO+KCl div 7-14	Two-way ANOVA and Bonferroni post hoc test
	> 0,9999	WT vs KO+KCl div 0-7	Two-way ANOVA and Bonferroni post hoc test
	0,0006	WT vs KO+KCl div 7-14	Two-way ANOVA and Bonferroni post hoc test
EV4 G	0,0488	WT vs KO	Two-way ANOVA and Bonferroni post hoc test
	> 0,9999	WT+KCL vs KO+KCl div 0-7	Two-way ANOVA and Bonferroni post hoc test
	0,0026	WT+KCL vs KO+KCl div 7-14	Two-way ANOVA and Bonferroni post hoc test
	0,9249	WT vs KO+KCl div 0-7	Two-way ANOVA and Bonferroni post hoc test
	> 0,9999	WT vs WT+ KCl div 7-14	Two-way ANOVA and Bonferroni post hoc test
**Figure S1**	**P value**	**Comparison**	**Statistical test**
S1 A	0,2573	WT vs WT+CX546 Div3-6 nº of intersections	One-way ANOVA and Tukey's post hoc test
	0,9012	WT vs WT+CX546 Div7-10 nº of intersections	One-way ANOVA and Tukey's post hoc test
S1 B	0,6597	WT vs WT+CX546 Div3-6 TDL	One-way ANOVA and Tukey's post hoc test
	0,0724	WT vs WT+CX546 Div7-10 TDL	One-way ANOVA and Tukey's post hoc test
S1 C	0,9356	WT vs WT+CX546 Div3-6 Max length	One-way ANOVA and Tukey's post hoc test
	0,5863	WT vs WT+CX546 Div7-10 Max length	One-way ANOVA and Tukey's post hoc test
S1 D	0,4155	WT vs WT+CX546 Div3-6 Polarization	One-way ANOVA and Tukey's post hoc test
	0,3252	WT vs WT+CX546 Div7-10 Polarization	One-way ANOVA and Tukey's post hoc test
S1 E	0,2214	WT vs WT+Cx546 Div3-6 Arc	One-way ANOVA and Dunnett's post hoc test
	0,679	WT vs WT+Cx546 Div3-6 Arhgdig	One-way ANOVA and Dunnett's post hoc test
	0,8696	WT vs WT+Cx546 Div3-6 Bdnf	One-way ANOVA and Dunnett's post hoc test
	0,9179	WT vs WT+Cx546 Div3-6 Cacna1d	One-way ANOVA and Dunnett's post hoc test

	0,5492	WT vs WT+Cx546 Div3-6 Camk2b	One-way ANOVA and Dunnett's post hoc test
	0,8461	WT vs WT+Cx546 Div3-6 Clcn3	One-way ANOVA and Dunnett's post hoc test
	0,0114	WT vs WT+Cx546 Div3-6 Dcx	One-way ANOVA and Dunnett's post hoc test
	0,741	WT vs WT+Cx546 Div3-6 Gabbr1	One-way ANOVA and Dunnett's post hoc test
	0,2076	WT vs WT+Cx546 Div3-6 Gabra3	One-way ANOVA and Dunnett's post hoc test
	0,0689	WT vs WT+Cx546 Div3-6 Gap43	One-way ANOVA and Dunnett's post hoc test
	0,9258	WT vs WT+Cx546 Div3-6 Gria2	One-way ANOVA and Dunnett's post hoc test
	0,1921	WT vs WT+Cx546 Div3-6 Grin1	One-way ANOVA and Dunnett's post hoc test
	0,3262	WT vs WT+Cx546 Div3-6 Grm5	One-way ANOVA and Dunnett's post hoc test
	0,4782	WT vs WT+Cx546 Div3-6 Kcc2	One-way ANOVA and Dunnett's post hoc test
	0,0581	WT vs WT+Cx546 Div3-6 Kcnq2	One-way ANOVA and Dunnett's post hoc test
	0,0053	WT vs WT+Cx546 Div3-6 Kcnq3	One-way ANOVA and Dunnett's post hoc test
	0,3467	WT vs WT+Cx546 Div3-6 Map2	One-way ANOVA and Dunnett's post hoc test
	0,3467	WT vs WT+Cx546 Div3-6 Neun	One-way ANOVA and Dunnett's post hoc test
	0,3467	WT vs WT+Cx546 Div3-6 Nr4a1	One-way ANOVA and Dunnett's post hoc test
	0,3467	WT vs WT+Cx546 Div3-6 Nurr1	One-way ANOVA and Dunnett's post hoc test
	0,3467	WT vs WT+Cx546 Div3-6 Psd95	One-way ANOVA and Dunnett's post hoc test
	0,3467	WT vs WT+Cx546 Div3-6 Scn2b	One-way ANOVA and Dunnett's post hoc test
	0,3467	WT vs WT+Cx546 Div3-6 Scn3b	One-way ANOVA and Dunnett's post hoc test
	0,3467	WT vs WT+Cx546 Div3-6 Scn8a	One-way ANOVA and Dunnett's post hoc test
	0,3467	WT vs WT+Cx546 Div3-6 Syn1	One-way ANOVA and Dunnett's post hoc test
	0,3467	WT vs WT+Cx546 Div3-6 Tuj1	One-way ANOVA and Dunnett's post hoc test

S1 F	0,7839	WT vs WT+Cx546 Div7-10 Arc	One-way ANOVA and Dunnett's post hoc test
	0,9912	WT vs WT+Cx546 Div7-10 Arhgdig	One-way ANOVA and Dunnett's post hoc test
	0,9903	WT vs WT+Cx546 Div7-10 Bdnf	One-way ANOVA and Dunnett's post hoc test
	0,8638	WT vs WT+Cx546 Div7-10 Cacna1d	One-way ANOVA and Dunnett's post hoc test
	0,9803	WT vs WT+Cx546 Div7-10 Camk2b	One-way ANOVA and Dunnett's post hoc test
	0,9819	WT vs WT+Cx546 Div7-10 Clcn3	One-way ANOVA and Dunnett's post hoc test
	0,7438	WT vs WT+Cx546 Div7-10 Dcx	One-way ANOVA and Dunnett's post hoc test
	0,9333	WT vs WT+Cx546 Div7-10 Gabbr1	One-way ANOVA and Dunnett's post hoc test
	0,5644	WT vs WT+Cx546 Div7-10 Gabra3	One-way ANOVA and Dunnett's post hoc test
	0,9969	WT vs WT+Cx546 Div7-10 Gap43	One-way ANOVA and Dunnett's post hoc test
	0,4102	WT vs WT+Cx546 Div7-10 Gria2	One-way ANOVA and Dunnett's post hoc test
	0,8126	WT vs WT+Cx546 Div7-10 Grin1	One-way ANOVA and Dunnett's post hoc test
	0,6001	WT vs WT+Cx546 Div7-10 Grm5	One-way ANOVA and Dunnett's post hoc test
	0,6001	WT vs WT+Cx546 Div7-10 Kcc2	One-way ANOVA and Dunnett's post hoc test
	0,5338	WT vs WT+Cx546 Div7-10 Kcnq2	One-way ANOVA and Dunnett's post hoc test
	0,9976	WT vs WT+Cx546 Div7-10 Kcnq3	One-way ANOVA and Dunnett's post hoc test
	0,5748	WT vs WT+Cx546 Div7-10 Map2	One-way ANOVA and Dunnett's post hoc test
	0,936	WT vs WT+Cx546 Div7-10 Neun	One-way ANOVA and Dunnett's post hoc test
	0,7794	WT vs WT+Cx546 Div7-10 Nr4a1	One-way ANOVA and Dunnett's post hoc test
	0,9964	WT vs WT+Cx546 Div7-10 Nurr1	One-way ANOVA and Dunnett's post hoc test
	0,9873	WT vs WT+Cx546 Div7-10 Psd95	One-way ANOVA and Dunnett's post hoc test
	0,9961	WT vs WT+Cx546 Div7-10 Scn2b	One-way ANOVA and Dunnett's post hoc test

	0,4494	WT vs WT+Cx546 Div7-10 Scn3b	One-way ANOVA and Dunnett's post hoc test
	0,0933	WT vs WT+Cx546 Div7-10 Scn8a	One-way ANOVA and Dunnett's post hoc test
	0,9841	WT vs WT+Cx546 Div7-10 Syn1	One-way ANOVA and Dunnett's post hoc test
	0,9924	WT vs WT+Cx546 Div7-10 Tuj1	One-way ANOVA and Dunnett's post hoc test
**Figure S3**	**P value**	**Comparison**	**Statistical test**
S3 A	>0,9999	WT vs WT+CX546	Student's t-test
S3 B	0,8796	WT vs WT+CX546 P30	Two-way ANOVA and Bonferroni post hoc test
	0,9271	WT vs WT+CX546 P50	Two-way ANOVA and Bonferroni post hoc test
	>0,9999	WT vs WT+CX546 P70	Two-way ANOVA and Bonferroni post hoc test
	0,071	WT vs WT+CX546 P90	Two-way ANOVA and Bonferroni post hoc test
S3 D	0,4483	WT vs WT+CX546	Student's t-test
S3 E	>0,9999	WT vs WT+CX546	Two-way ANOVA and Bonferroni post hoc test
	>0,9999	WT vs KO	Two-way ANOVA and Bonferroni post hoc test
	>0,9999	WT vs KO+CX546	Two-way ANOVA and Bonferroni post hoc test
S3 F	0,82	WT vs WT+CX546	Student's t-test
S3 G	0,0754	WT vs WT+CX546	Student's t-test
S3 H	0,201	WT vs WT+CX546	Student's t-test

Appendix Figure S1:

A-D: Scatter plots showing the effects of early and late CX546 treatments on 4 morphological parameters analyzed in wt cultures. One-way ANOVA highlighted no difference between groups. Values represent the mean ± SEM. Sample size: n≥12 wells deriving from three independent preparations, for each well at least 15 cells were measured.

E,F: Scatter plots showing the effect of early and late CX546 treatments on the expression levels of 27 neuronal genes in wt cultures. One-way ANOVA highlighted no difference between groups. Values represent the mean ± SEM. Sample size: n≥8 wells deriving from two independent preparations.

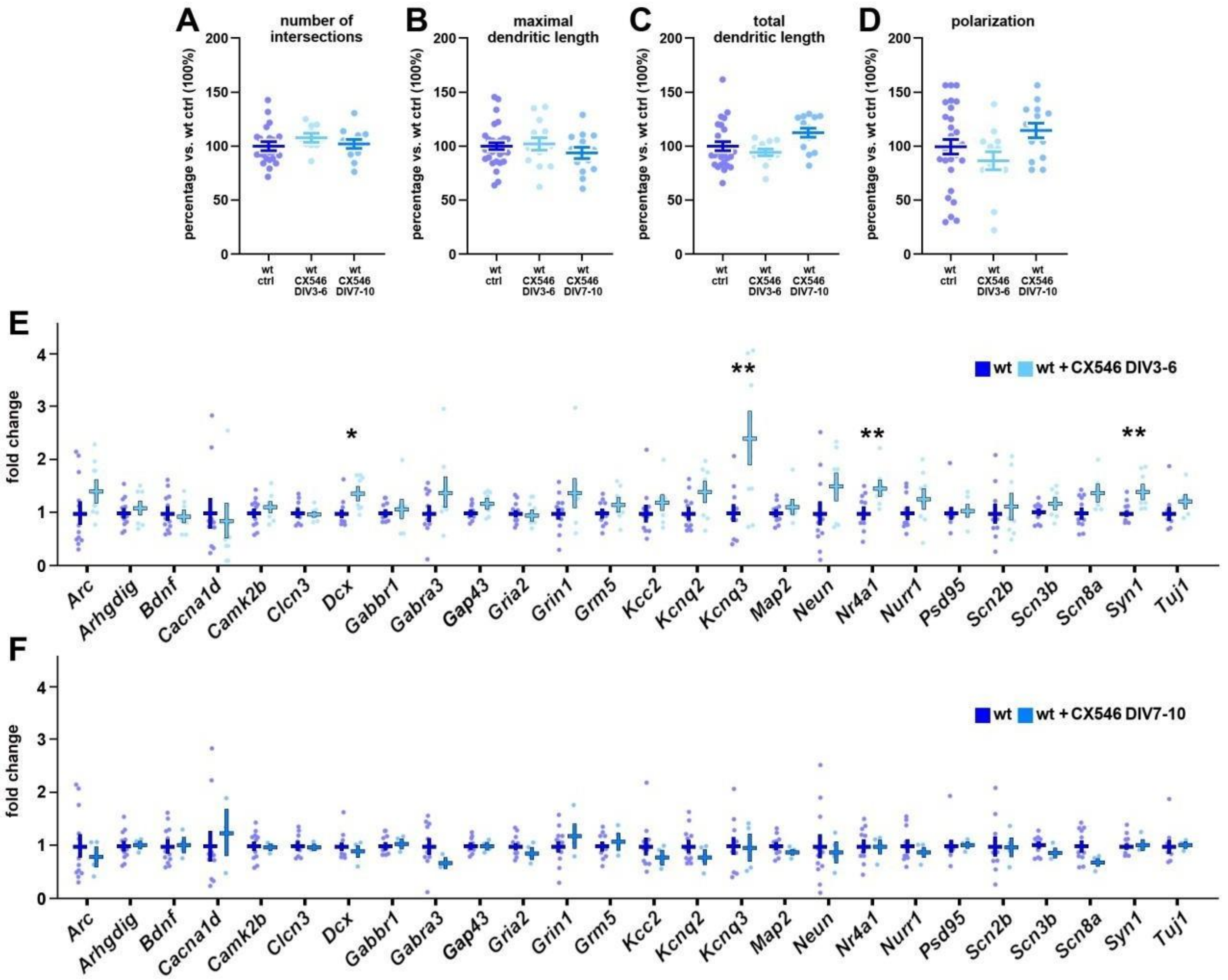

Appendix Figure S2:

A,B: Two-way ANOVA followed by Bonferroni *post hoc* test, in which variables were batch and genotype, was used to highlight and discard batch effects. Our statistical approach identified 43 transcripts that survived the statistical cut-off, while only 8 were discarded (in gray). Significant p-values (<0,05) are depicted in red. Each bar of the graph (B) represents the average expression levels for each gene. Histograms show the mean ± SEM. *: p-value<0.05; **: p-value<0.01; ***: p-value<0.001. Sample size: n≥18 samples deriving from three independent preparations.

A p-values

name	batch	genotype
Adcy9	0.033	0.373
Arc	0.486	0.050
Arhgdig	0.786	0.030
Bdnf	0.188	0.004
Bhlhb5	0.841	0.002
Cacna1d	0.733	0.086
Camk2b	0.085	0.000
Camk2d	0.211	0.722
Clcn3	0.239	0.032
Clcn7	0.209	0.826
Cux1	0.492	0.109
Dcx	0.002	0.001
Dusp	0.557	0.685
Egr1	0.057	0.091
Fezf2	0.037	0.059
Gabbr1	0.489	0.000
Gabra3	0.760	0.000
Gad67	0.005	0.059
Gap43	0.735	0.000
Gnaq	0.403	0.371
Gria1	0.016	0.627
Gria2	0.254	0.002
Gria3	0.029	0.706
Gria4	0.202	0.817
Grin1	0.131	0.004
Grin2b	0.000	0.700
Grin2c	0.678	0.697
Grin2d	0.021	0.365
Grm3	0.062	0.619
Grm5	0.003	0.000
Grm7	0.089	0.322
Jph1	0.472	0.744
Kcc2	0.140	0.000
Kcnq2	0.111	0.001
Kcnq3	0.650	0.087
Map2	0.582	0.002
Necab1	0.172	0.269
Neun	0.117	0.016
Nkcc1	0.616	0.121
Nr4a1	0.067	0.003
Nurr1	0.671	0.040
Plgc2	0.290	0.721
Ppp1r3c	0.042	0.476
Psd95	0.157	0.004
Satb2	0.546	0.069
Scn2b	0.303	0.005
Scn3b	0.001	0.000
Scn8a	0.154	0.025
Syn1	0.002	0.000
Tuj1	0.001	0.000

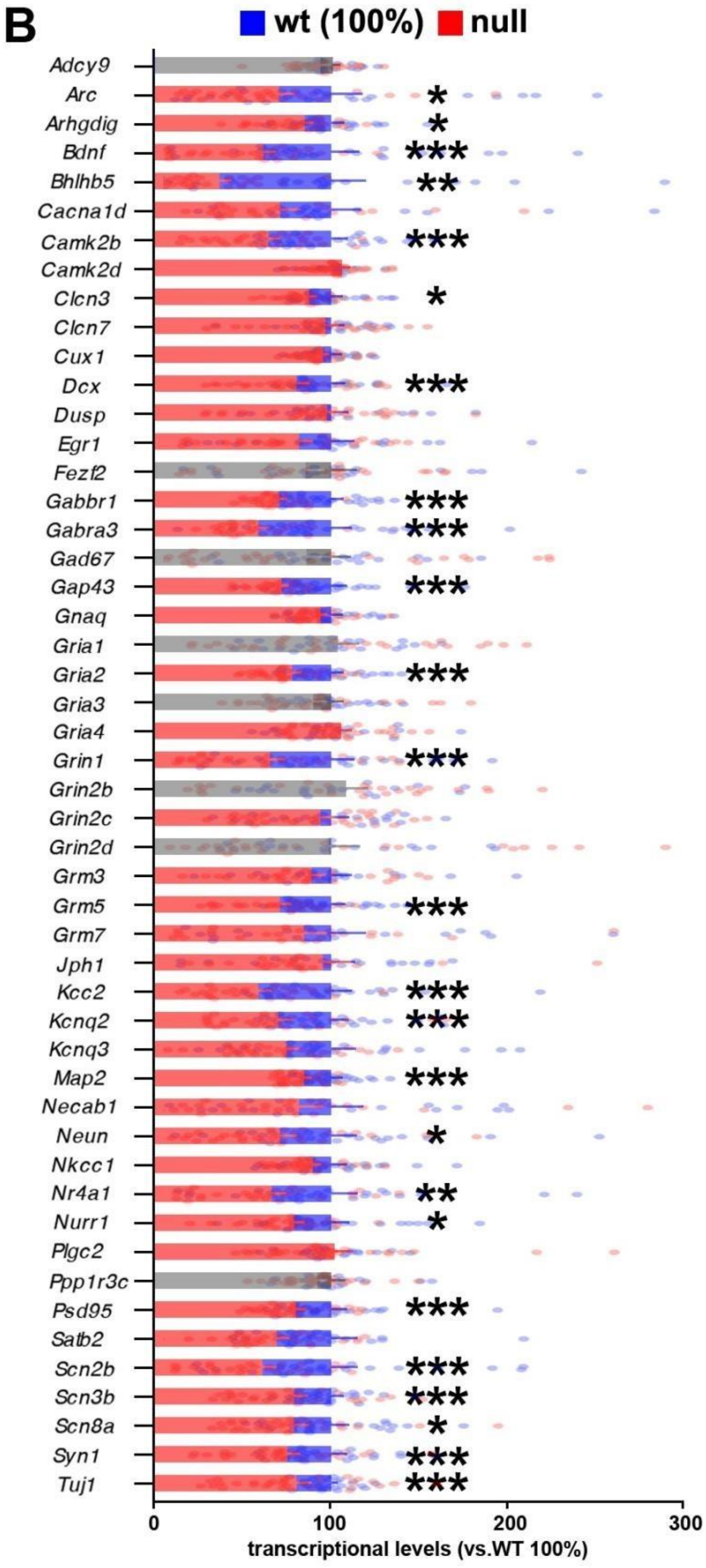

Appendix Figure S3:

A: Kaplan-Mayer showing life span of wt mice treated with vehicle (blue) or with CX546 (light blue).

B: Scatter plots showing body weight of wt mice treated with vehicle or CX546 assessed at four different time points (P30, P50, P70 and P90). Two-Way ANOVA ($F(3,75)=232,4; p<0,0001$) indicates a significant effect of time but not of treatment. Sample size: n=22 (10 treated with vehicle and 12 treated with CX546).

C-c': The score for hind limbs clasping was assessed twice a week starting from P30 in wt mice treated with vehicle and wt mice treated with CX546. In c' severity scores were calculated at three different time points (P55, P70, P80). Sample size: n=22 (10 treated with vehicle and 12 treated with CX546).

D: Scatter plot showing latency to fall on Rotarod of wt mice treated with vehicle or CX546. Student's t-test highlighted no difference between groups. Sample size: n=15 (6 treated with vehicle and 9 treated with CX546). Each dot represents a single animal.

E: Scatter plot showing the time spent by mice exploring the two objects during the third day of test (depicted as %). Two-Way ANOVA did not indicate any significant effect of both genotype and treatment. Since all mice showed an exploration rate higher than 10% (depicted as dotted line) of the total time, they were all included in the Discrimination index assessment (see Materials and Methods). Data are represented as values ± SEM. Sample size: n=16 wt (8 treated with vehicle and 8 treated with CX546) and n=12 ko (6 treated with vehicle and 6 treated with CX546). Each dot represents a single animal.

F: Scatter plot showing the assessment of the discrimination index (DI) between two different objects for each mouse during the last trial of a Novel Object Recognition test in wt mice treated with vehicle or CX546. Student's t-test highlighted no significant difference between groups. Sample size: n=16 wt (8 treated with vehicle and 8 treated with CX546). Each dot represents a single animal.

G,H: Expression level of *Bdnf* and *Kcc2* measured in cortical samples of P45 wt mice treated with vehicle or CX546. Student's t-test highlighted no significant difference between groups. Sample size: n=15 wt (10 treated with vehicle and 5 treated with CX546). Each dot represents a single animal.

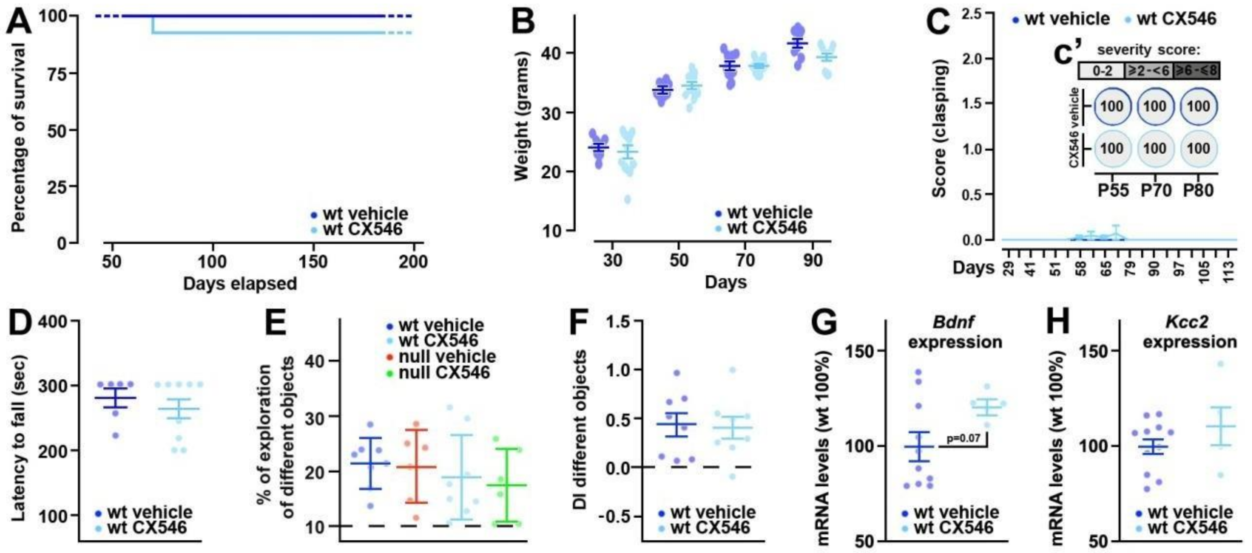

Supplement: Supplementary file 1 — Appendix [file EMMM-13-e12433-s005.pdf]
