# Supplementary material for: The enhancement of activity rescues the establishment of Mecp2 null neuronal phenotypes
Source: EMBO Mol Med. 2021 Mar 5;13(4):e12433. doi: 10.15252/emmm.202012433 (PMC8033520; doi:10.15252/emmm.202012433)
Supplement: Supplementary file 2 — Expanded View Figures PDF [file EMMM-13-e12433-s003.pdf]

Expanded View Figures

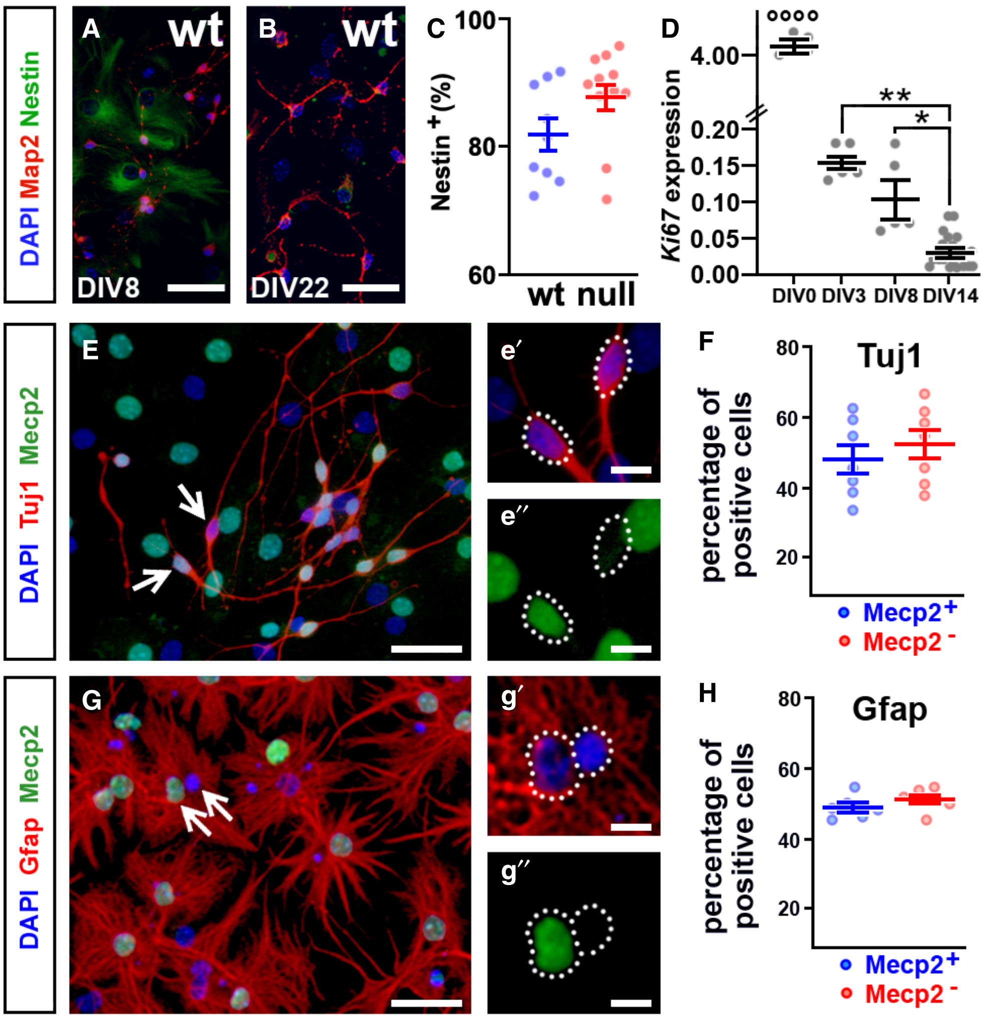

Figure EV1. Lack of Mecp2 does not influence the NPC fate commitment.

A–D DIV8 and DIV22 cultures were stained with Nestin (to mark neuroprogenitors; green) and Map2 (to mark neurons; red) (panels A and B). Scatter plot in C represents the number of Nestin-positive cells counted in both wt and null cultures at DIV8 (panel C). Scatter plot in D reports *Ki67* (marker of cell cycle) expression levels ($2^{-\Delta ct}$) at different DIVs. Panel C: Student's *t*-test, Panel D: two-way ANOVA and Bonferroni *post hoc* test. **P*-value < 0.05; ***P*-value < 0.01; °°°°*P*-value < 0.001. Scatter plots show the mean ± SEM from at least 6 wells obtained from two independent preparations. Scale bars A, B: 20 μm.

E–H Nuclei deriving from heterozygous cortices were stained for Mecp2 (green) and DAPI; neurons and astrocytes were distinguished using antibodies against Tuj1 and Gfap, respectively (red; E, G). White arrows indicate two representative cells that are positive or negative for Mecp2. Scatter plots report the percentage of cells positive or negative for Mecp2 counted at DIV22 over the total of Tuj1 (F) and Gfap (H) positive cells. No difference between the two groups was highlighted (Student's *t*-test). Graphs show the mean ± SEM from at least 3 wells obtained from two independent preparations; at least 10 fields were measured for each well. Scale bars: 30 μm (E, G); 10 μm (e′, e″, g′, g″).

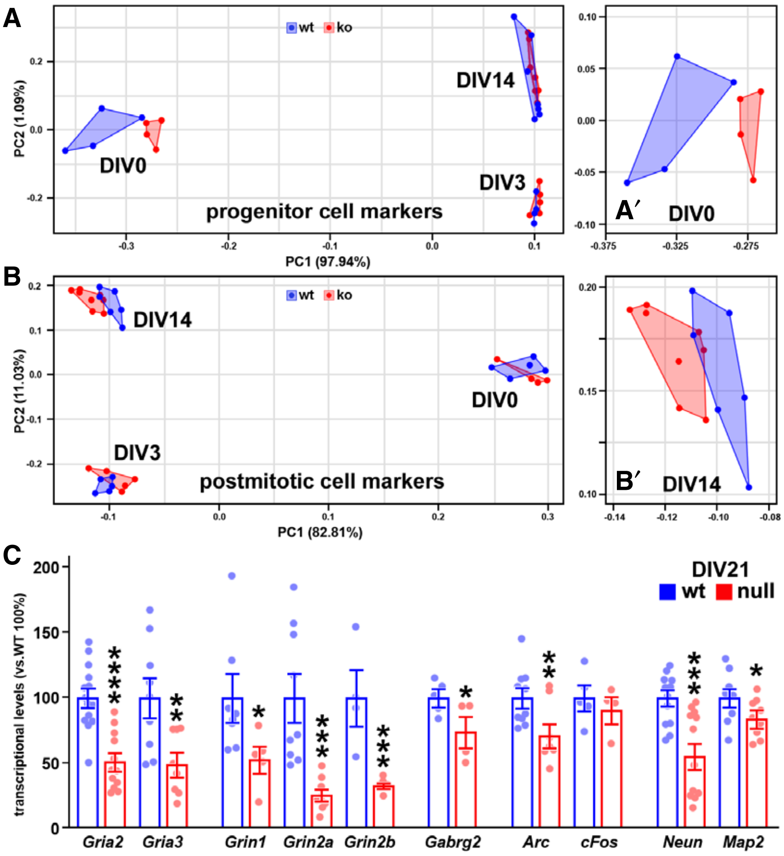

Figure EV2. Transcriptional differences between wt and *Mecp2* null samples in basal condition.

A, B PCA analyses show the segregation of wt and null samples based on the expression of different sets of genes (see Appendix Table S1). 16 genes typically expressed by cortical neuroprogenitors were considered in A, while 51 genes typically expressed by neurons were considered in B. A' and B' are close ups at different DIVs. At least 5 samples derived from three independent preparations were used to produce the points composing the wt and null cluster. Each point represents a single well.

C The expression of 10 neuronal genes was assessed by qRT–PCR at DIV21 in wt and *Mecp2* null cultures. Significant differences in gene expression between wt and null cultures were screened through Student's *t*-test. Histograms show the mean ± SEM from at least 3 samples obtained from two independent preparations. *P-value < 0.05; **P-value < 0.01; ***P-value < 0.001.

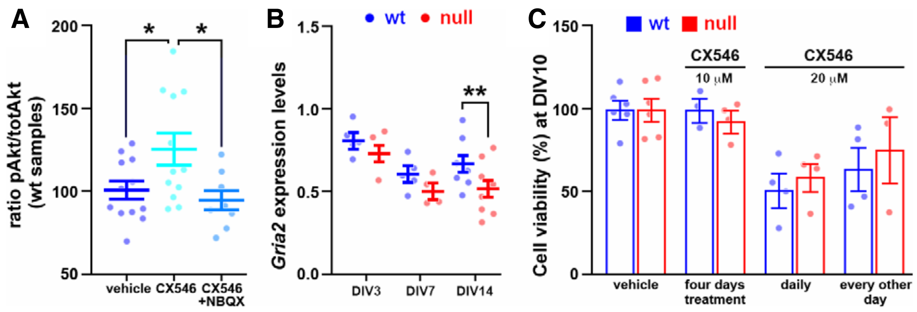

Figure EV3. Experimental setting of the *in vitro* Ampakine treatment.

A A scatter plot shows the levels of Akt phosphorylation in wt cells at DIV9 treated acutely with vehicle, CX546 or CX546 and NBQX. One-way ANOVA was used to assess difference between groups. Tukey's multiple comparison test highlighted significant differences between vehicle and CX546 treated cells and between CX546 treated cells and CX-NBQX treated cells. Values represent the mean ± SEM. *: P-value < 0.05. Sample size: at least 6 samples obtained from two independent preparations.

B Scatter plot shows the expression of the AMPA receptor subunit *Gria2* mRNA in cultures differentiated from NPCs at three different time points expressed as $2^{-\Delta ct}$ (DIV3, DIV7, and DIV14). Two-way ANOVA followed by Bonferroni *post hoc* test indicated a reduced *Gria2* transcription in null neurons only at DIV14. **: P-value < 0.01. Values represent the mean ± SEM. Sample size: 4 wells obtained from two independent preparations.

C To assess CX546 toxicity we measured cell viability through MTT assay. Cells were collected at DIV14 after Ampakine exposure (10 and 20 μM) from DIV7 to DIV10 and at DIV10 after daily or every other day exposure to 20 μM CX546. Two-way ANOVA indicated a significant effect of both the 20 μM exposures ($F(2, 17) = 8.913$ and $P = 0.0023$), thus indicating the toxicity of this protocol. Sample size: $n \geq 3$ wells deriving from two independent preparations. Values represent the mean ± SEM.

Source data are available online for this figure.

 © 2021 The Authors

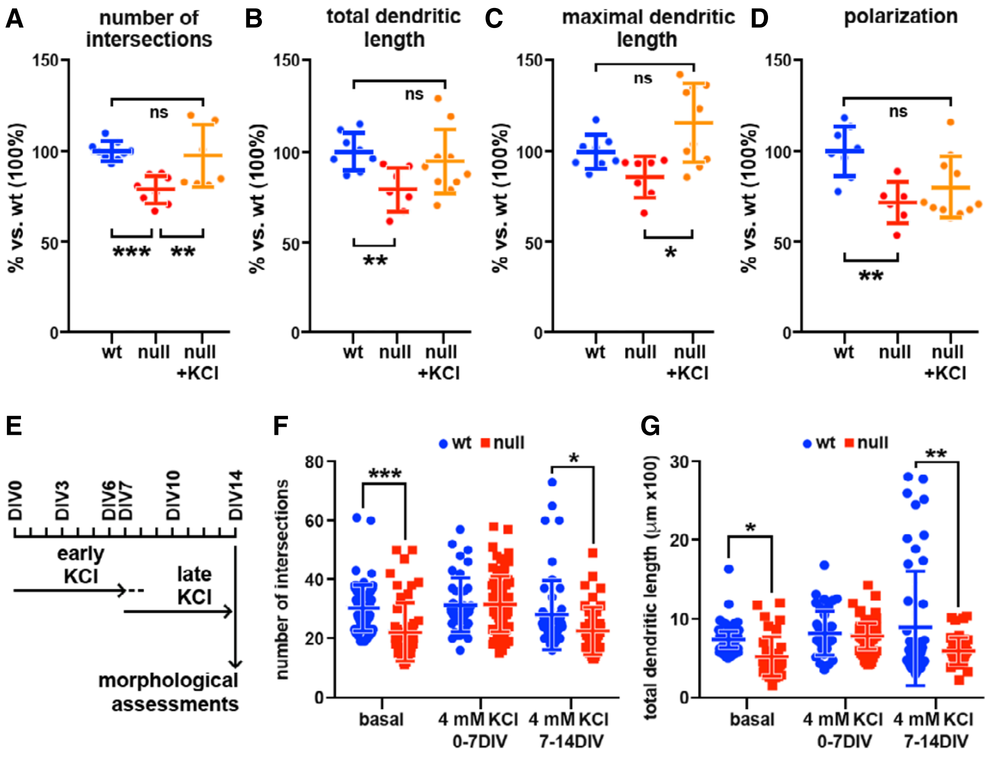

Figure EV4. KCl neuronal activity enhancement rescues *Mecp2* null defective morphology.

A-D Scatter plots showing the effects of adding 4 mM KCl on 4 morphological parameters assessed in wt and ko cultures at DIV22. One-way ANOVA followed by Tukey's multiple comparison test was used to assess differences between groups (*P-value < 0.05; **P-value < 0.01; ***P-value < 0.001). Values represent the mean ± SEM. Sample size: $n \geq 7$ wells from three independent preparations, at least 105 cells were measured in each well.

E Schematic representation of KCl exposure.

F, G Scatter plots showing the effects of an early (DIV0-7) and late (DIV7-14) treatment with 4 mM KCl on 2 morphological parameters assessed at DIV14 in wt and ko cultures. Sample size: $n \geq 40$ neurons from two independent preparations. Two-way ANOVA followed by Bonferroni multiple comparison test was used to assess differences between groups. (*P-value < 0.05; **P-value < 0.01; ***P-value < 0.001). Values represent the mean ± SEM.

© 2021 The Authors
