# Supplementary material for: The enhancement of activity rescues the establishment of Mecp2 null neuronal phenotypes
Source: EMBO Mol Med. 2021 Mar 5;13(4):e12433. doi: 10.15252/emmm.202012433 (PMC8033520; doi:10.15252/emmm.202012433)

Expanded View figure 3B

WB for p-Akt/Akt ratio on neuronal proteins

Loading samples: **WT- WT**+CX546-**WT**+CX546+Nbqx-**WT-WT**+CX546-**WT**+CX546+Nbqx-**WT-WT**+CX546-**WT**+CX546+Nbqx**-WT**

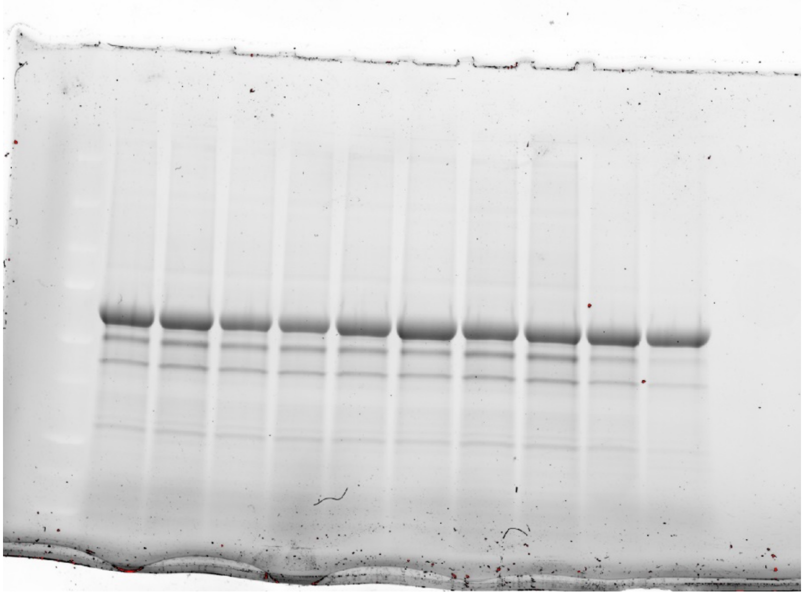

Marker

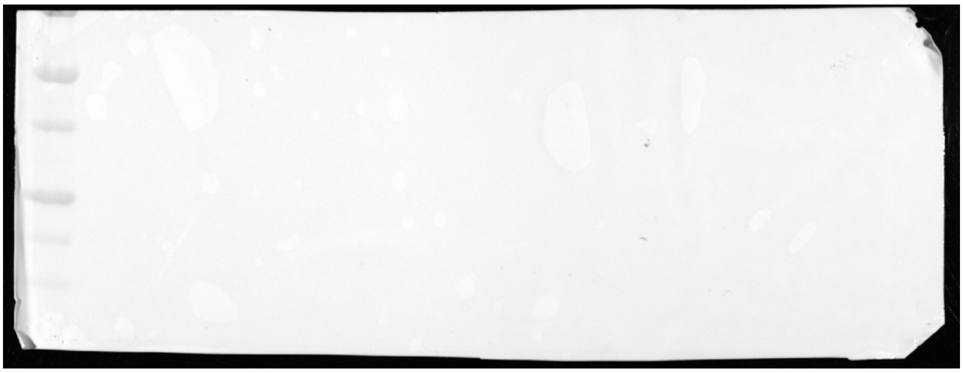

p-Akt

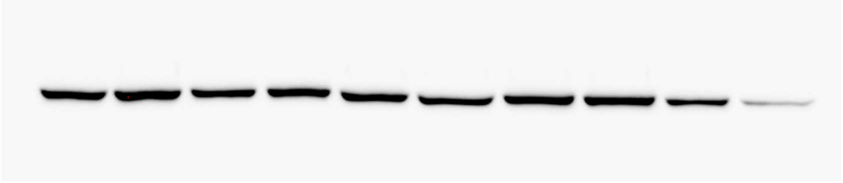

Akt tot

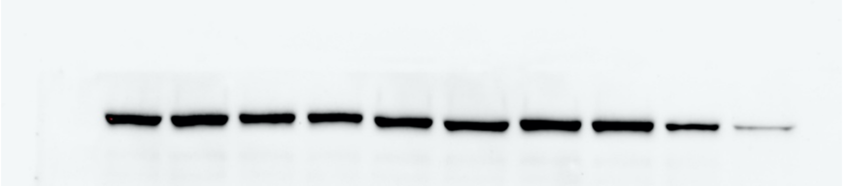

Supplement: Supplementary file 3 — Source Data for Expanded View [file EMMM-13-e12433-s001.zip › emmm202012433-sup-0004-SDataFigEV3B.PDF]
