# Supplementary material for: The enhancement of activity rescues the establishment of Mecp2 null neuronal phenotypes
Source: EMBO Mol Med. 2021 Mar 5;13(4):e12433. doi: 10.15252/emmm.202012433 (PMC8033520; doi:10.15252/emmm.202012433)

Figure 1 panel B-C

Tuj1-dapi    WT DIV 8

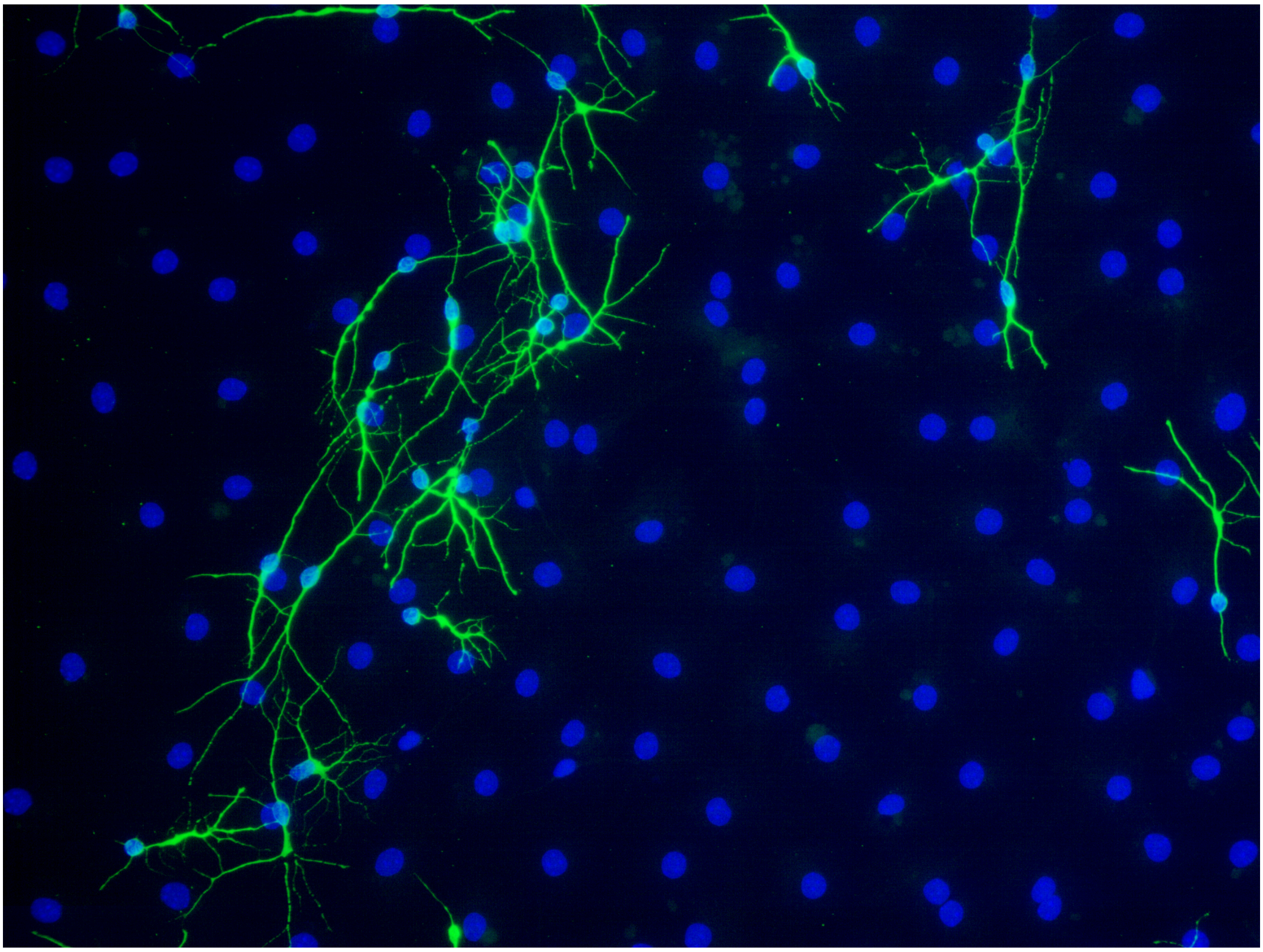

Tuj1-dapi    WT DIV 22

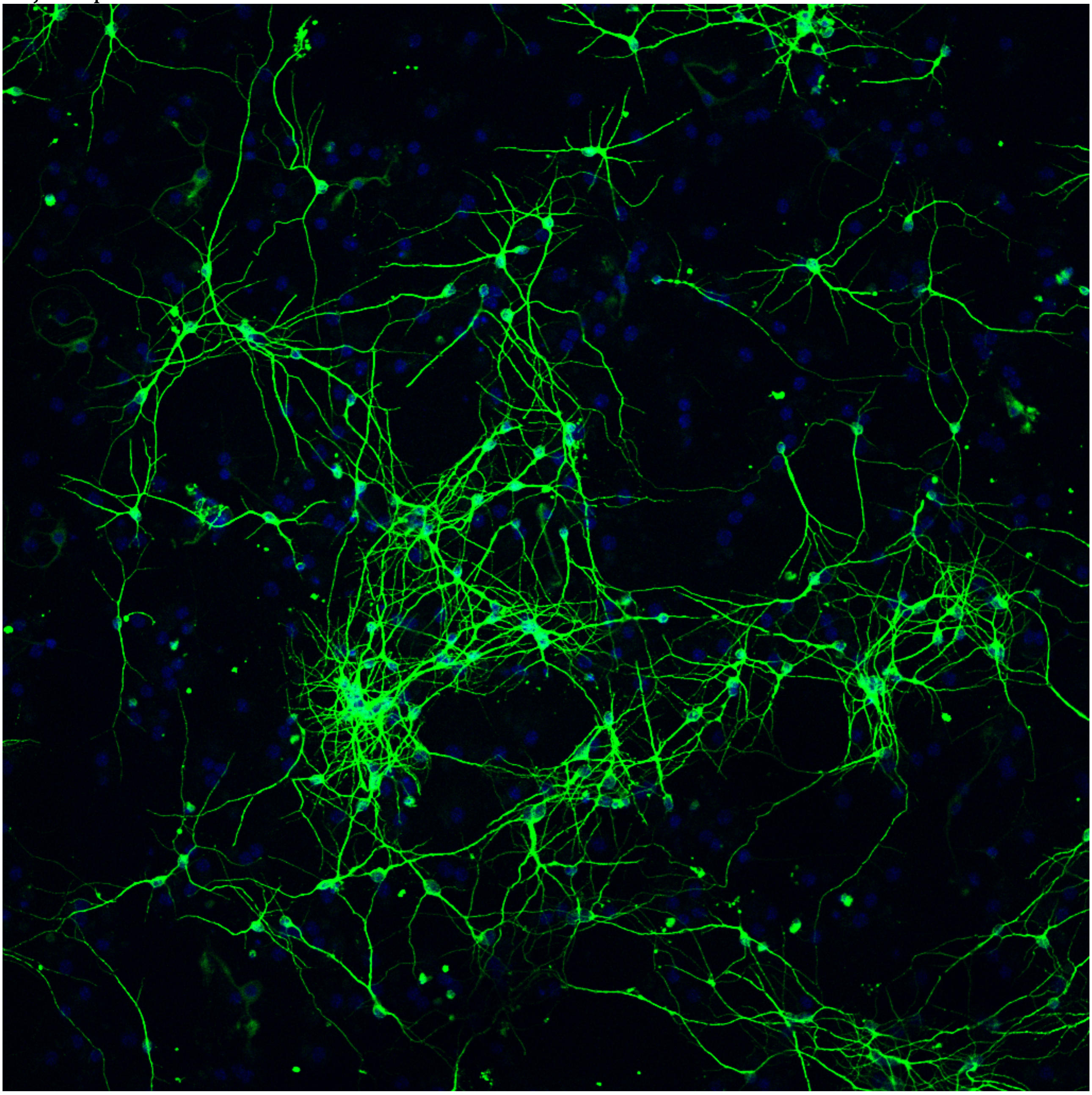

Tuj1-dapi   KO DIV8

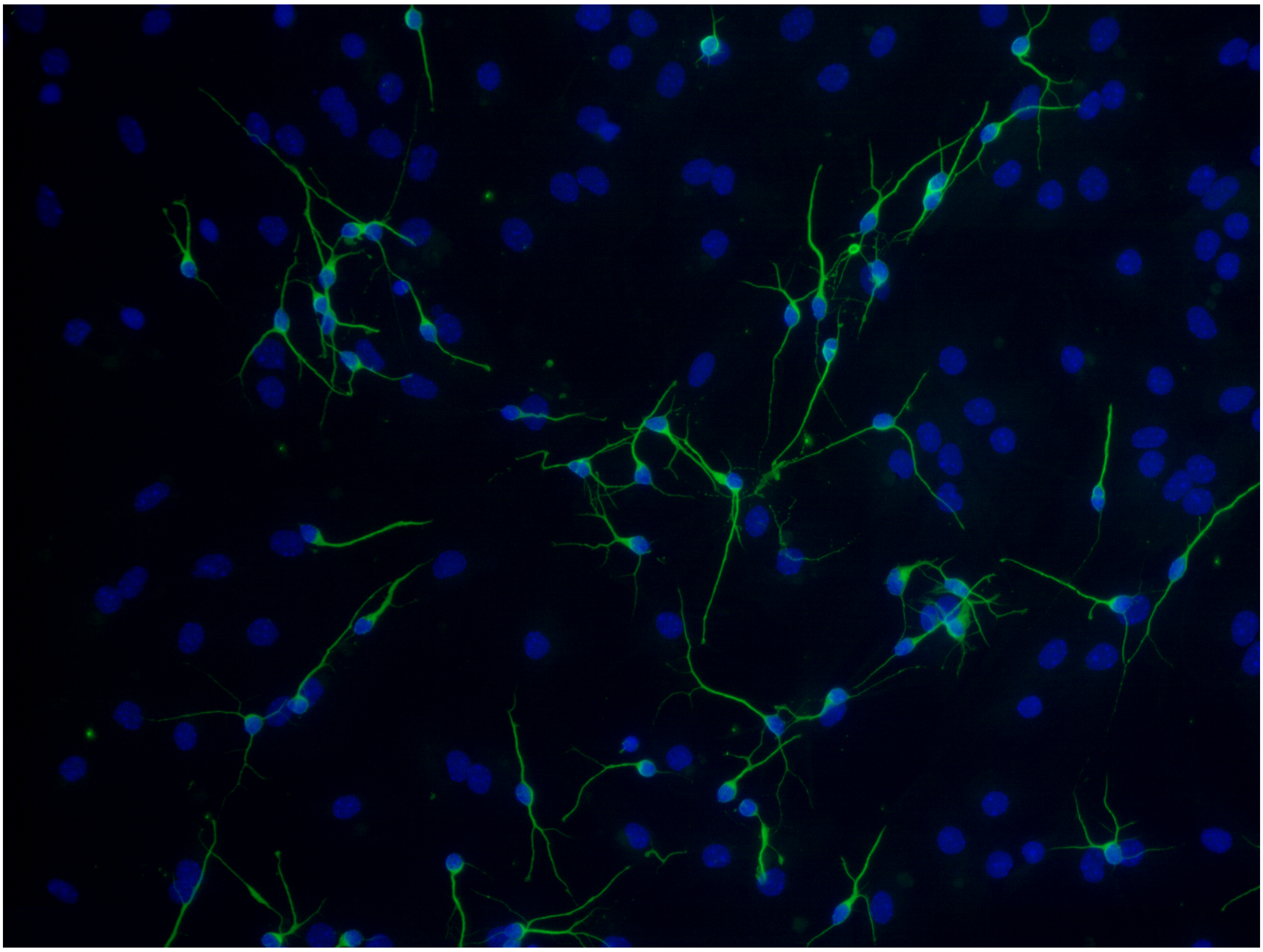

Tuj1-dapi   KO DIV22

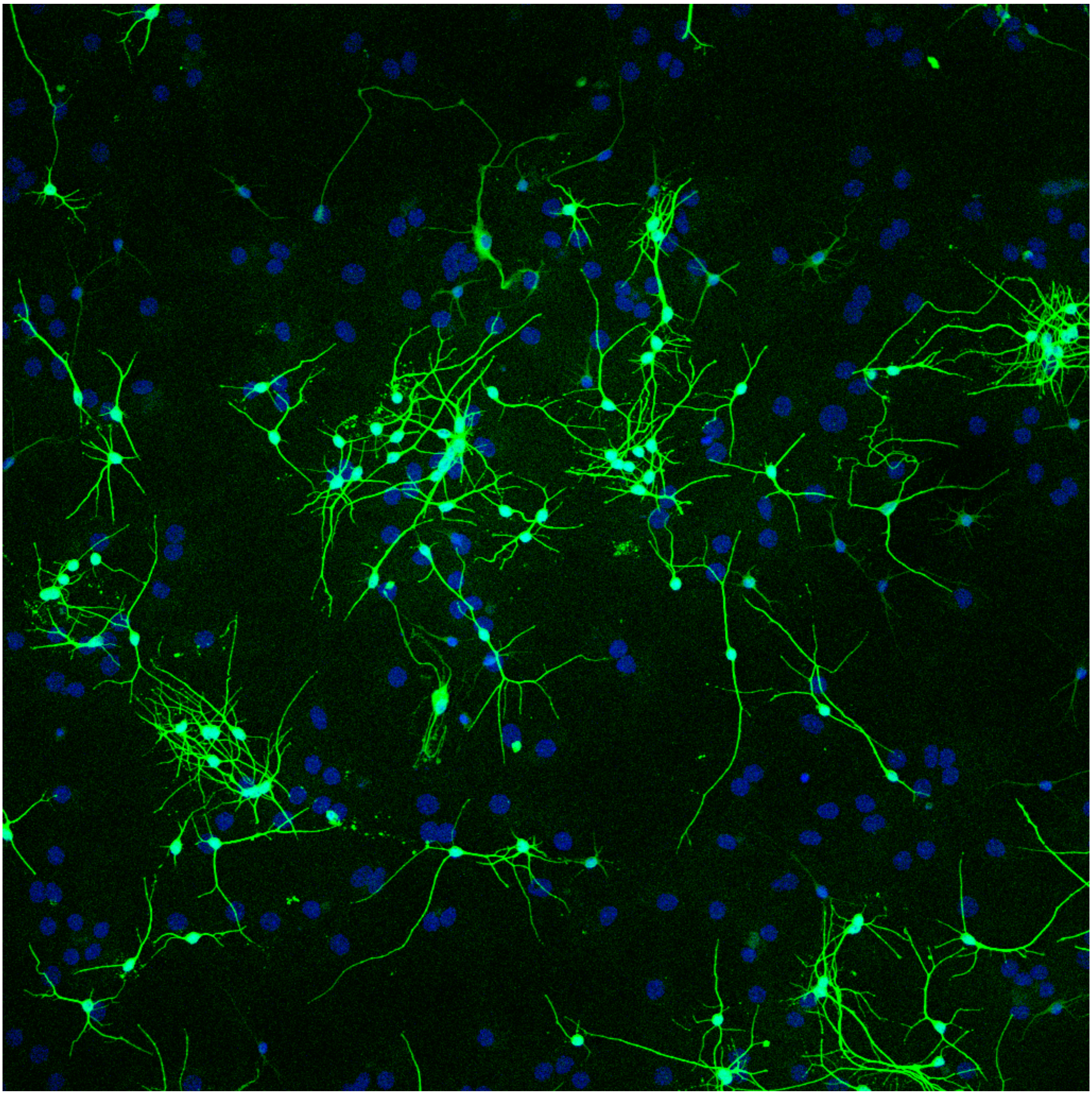

Figure 1 panel E-F

Gfap-dapi WT DIV8

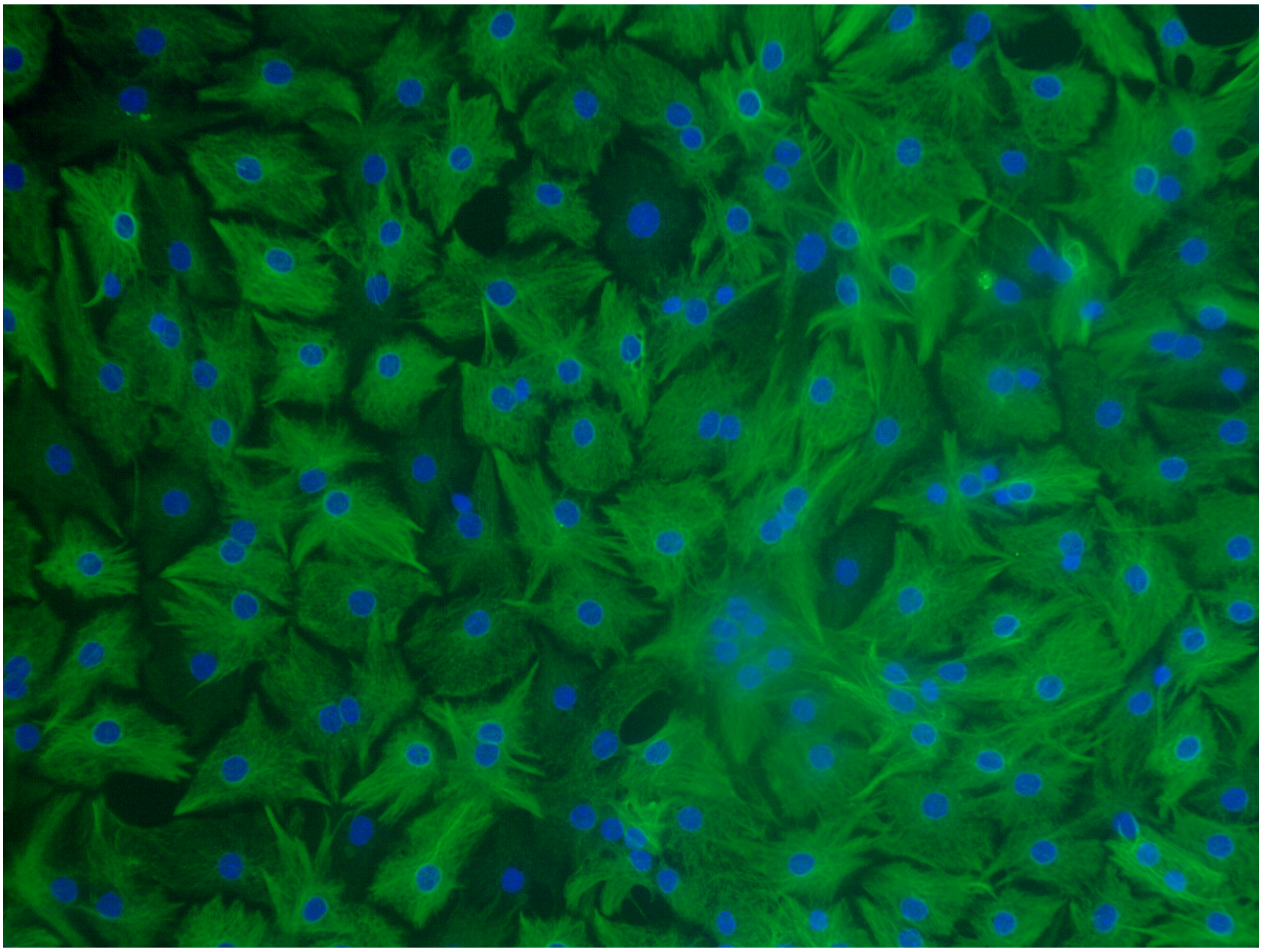

Gfap-dapi  WT DIV22

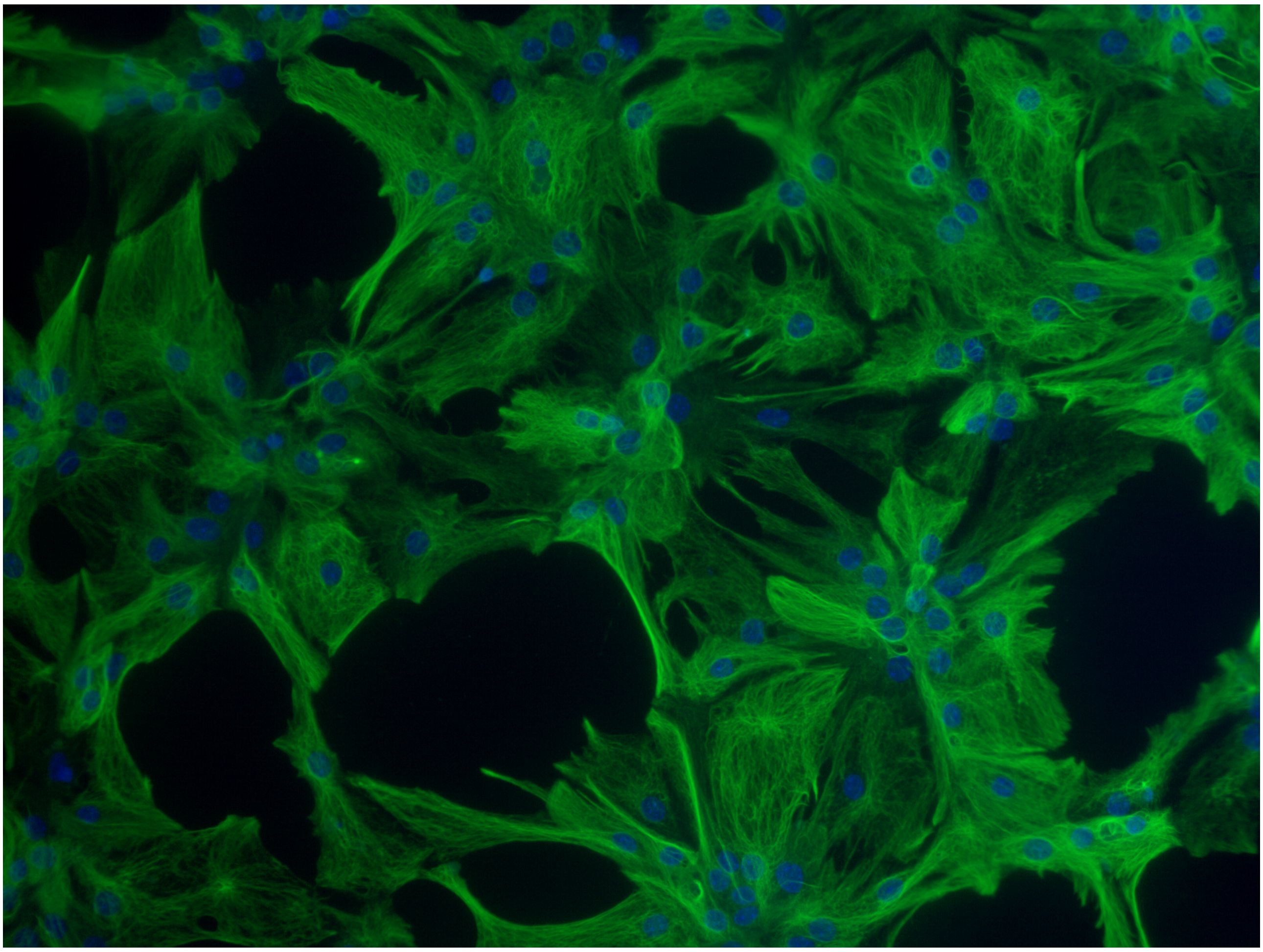

Gfap-dapi  KO DIV8

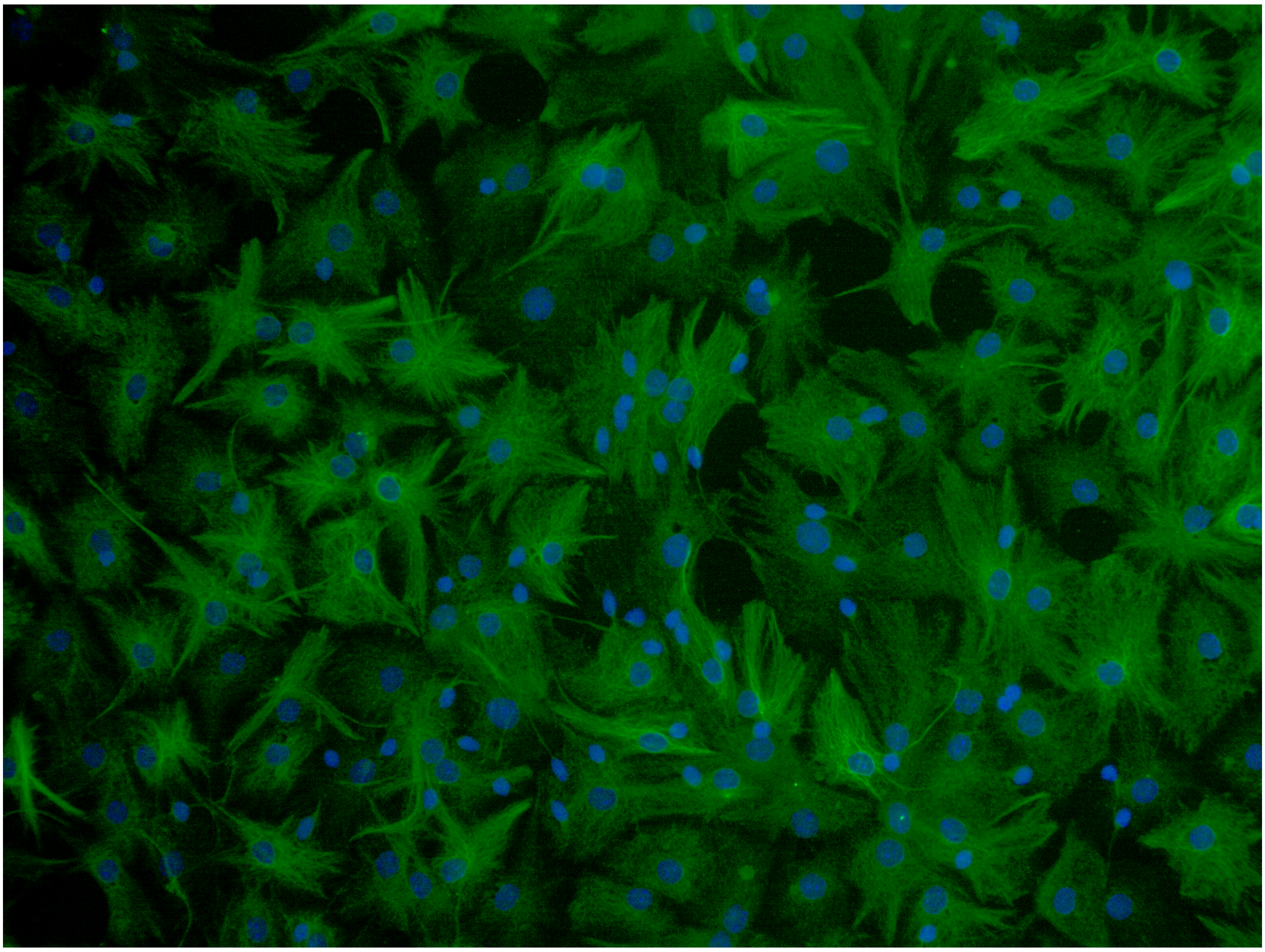

Gfap-dapi  DIV22

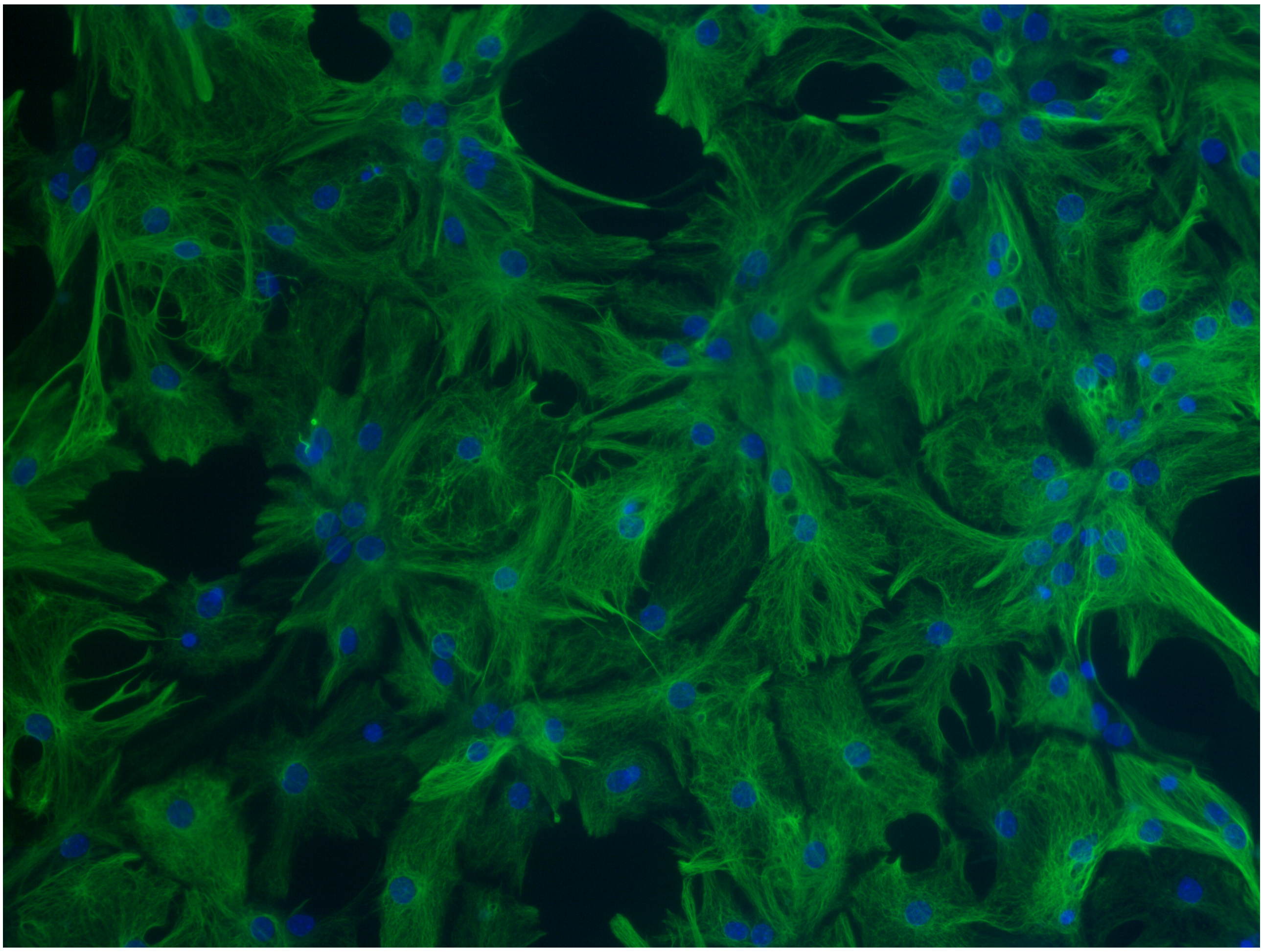

Figure 1 panel H-I

Olig2-dapi WT DIV8

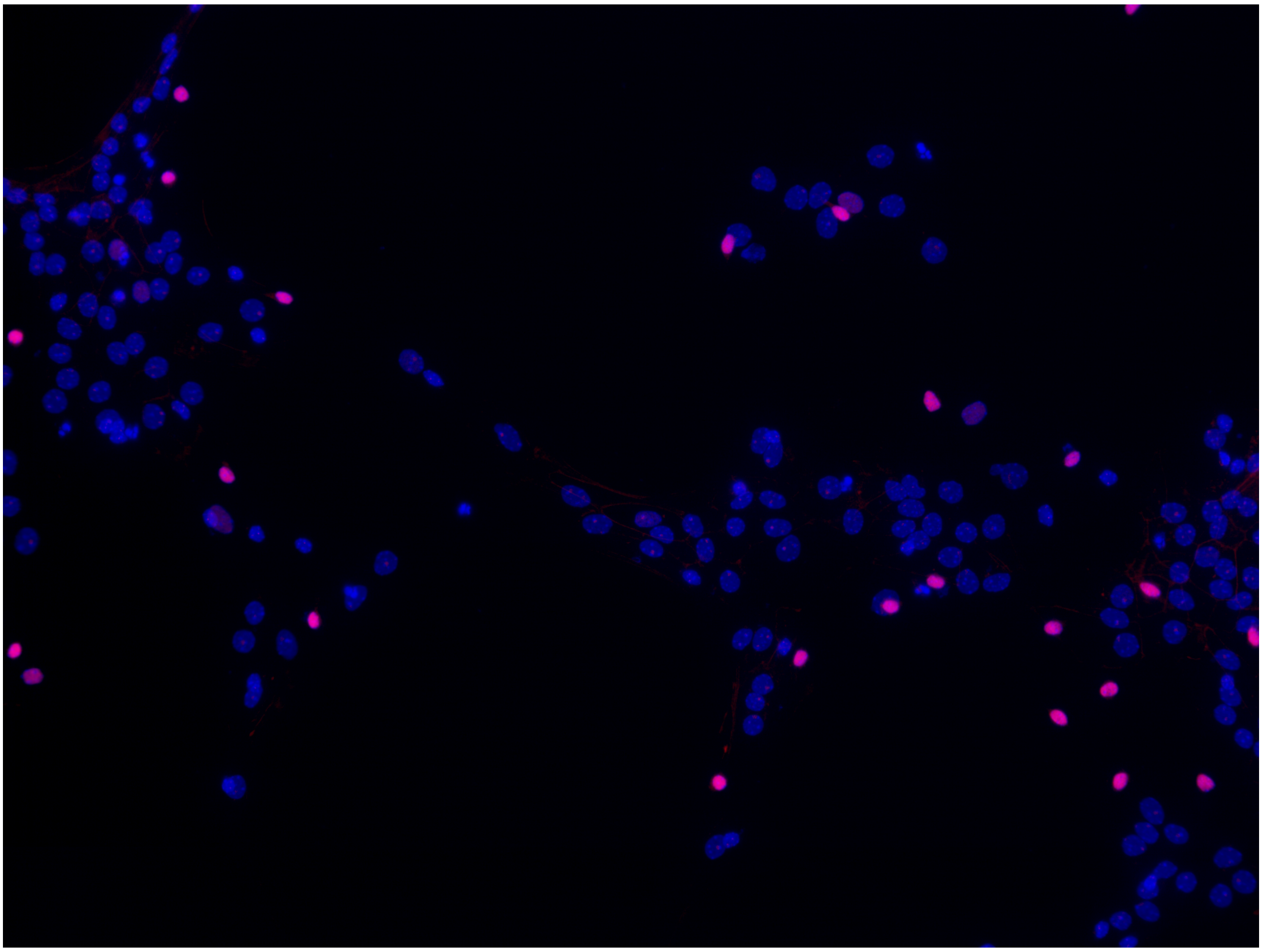

Olig2-dapi  WT DIV22

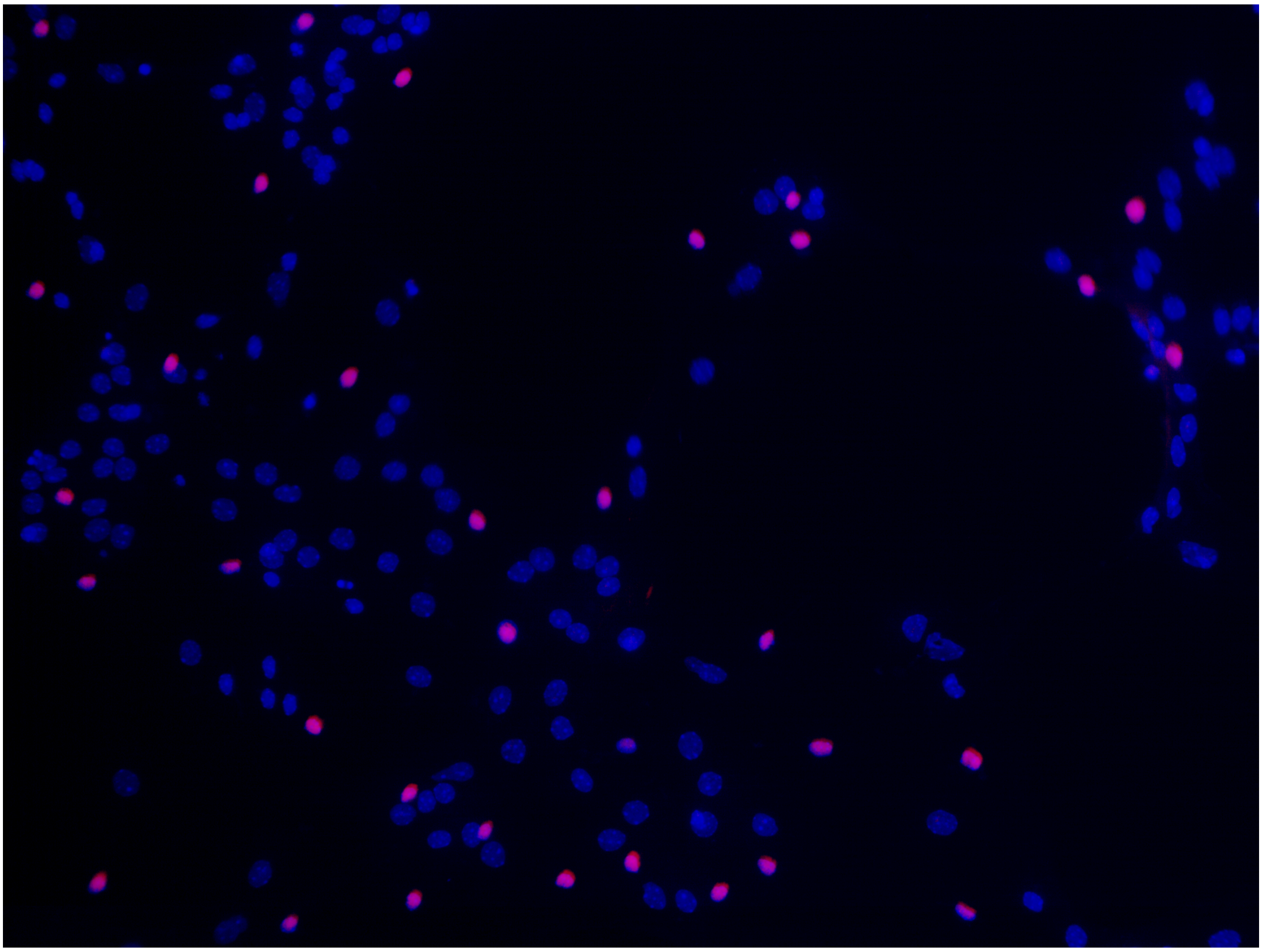

Olig2-dapi  KO DIV 8

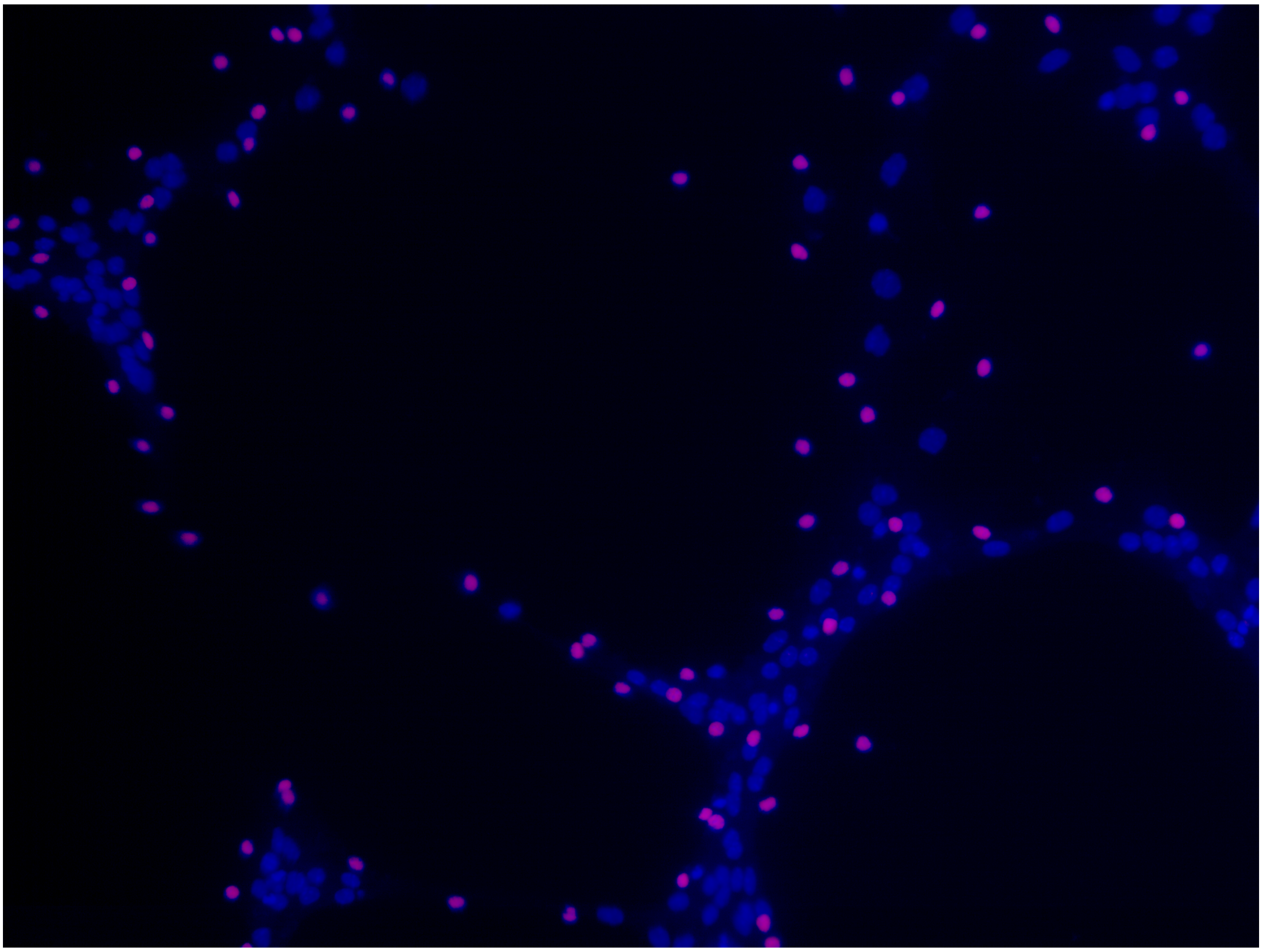

Olig2-dapi  KO DIV22

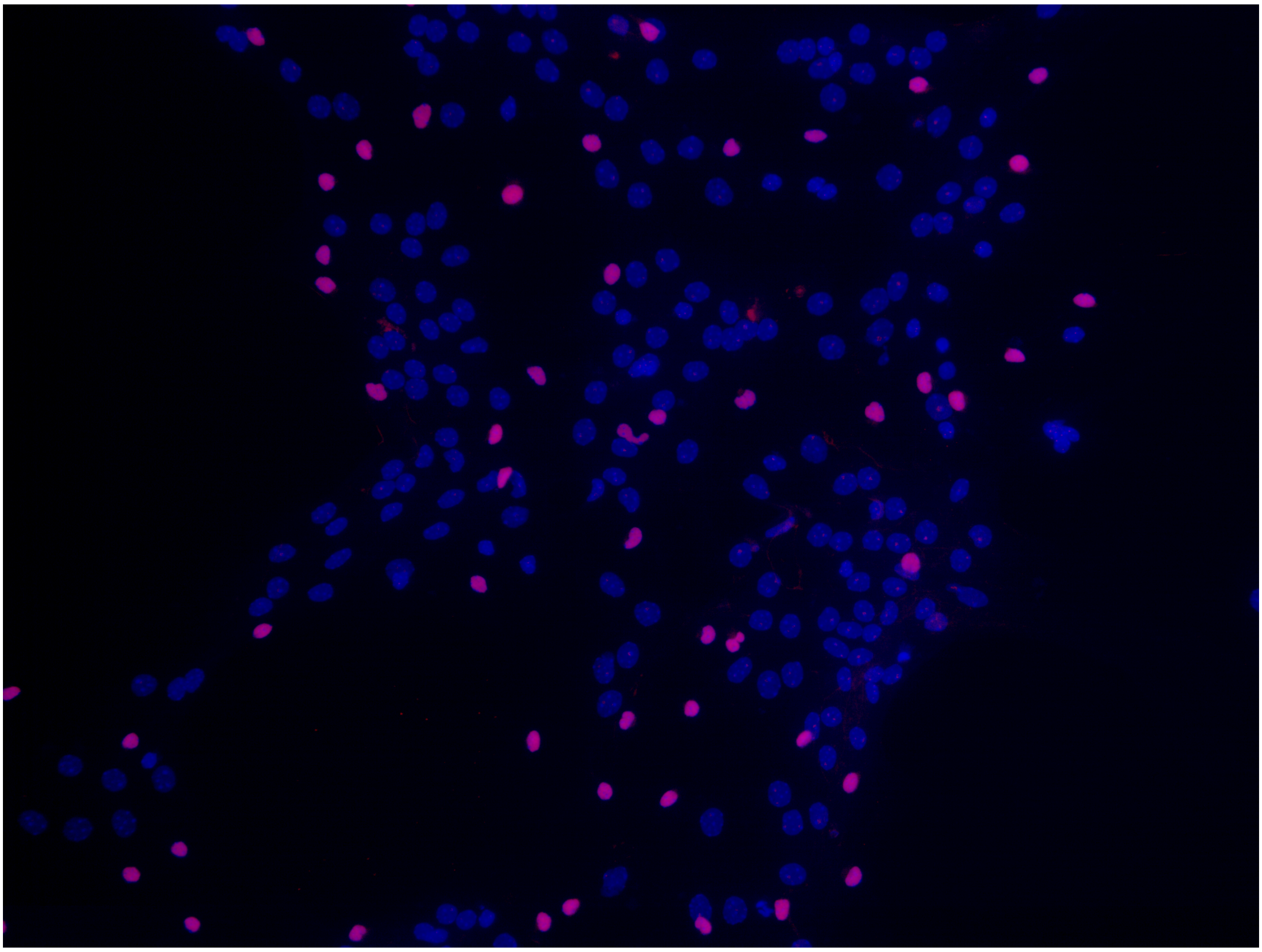

Figure 1 panel K

Tuj1(green)-Neun (red)-dapi  WT DIV14

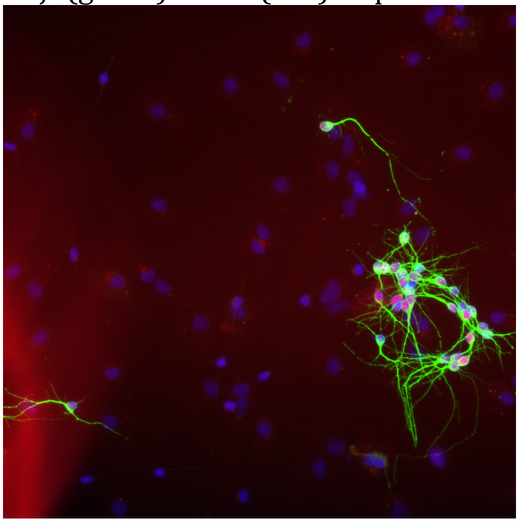

Tuj1(green)-Neun (red)-dapi  KO DIV14

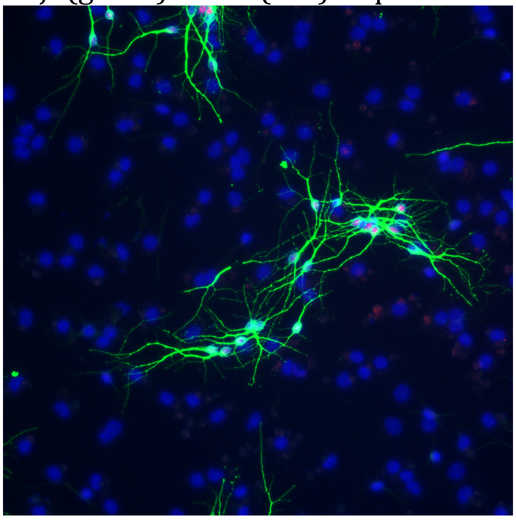

Supplement: Supplementary file 5 — Source Data for Figure 1 [file EMMM-13-e12433-s004.pdf]
